# Thuwalallenes A–E and Thuwalenynes A–C: New C_15_ Acetogenins with Anti-Inflammatory Activity from a Saudi Arabian Red Sea *Laurencia* sp.

**DOI:** 10.3390/md17110644

**Published:** 2019-11-15

**Authors:** Aikaterini Koutsaviti, Maria G. Daskalaki, Susana Agusti, Sotirios C. Kampranis, Christos Tsatsanis, Carlos M. Duarte, Vassilios Roussis, Efstathia Ioannou

**Affiliations:** 1 Section of Pharmacognosy and Chemistry of Natural Products, Department of Pharmacy, National and Kapodistrian University of Athens, Panepistimiopolis Zografou, 15771 Athens, Greece; kkoutsaviti@pharm.uoa.gr (A.K.); roussis@pharm.uoa.gr (V.R.); 2 Laboratory of Clinical Chemistry, School of Medicine, University of Crete, 70013 Heraklion, Greece; m.daskalaki@med.uoc.gr (M.G.D.); soka@plen.ku.dk (S.C.K.); tsatsani@uoc.gr (C.T.); 3 Red Sea Research Center, King Abdullah University of Science and Technology, Thuwal 23955-6900, Saudi Arabia; susana.agusti@kaust.edu.sa (S.A.); carlos.duarte@kaust.edu.sa (C.M.D.); 4 Institute of Molecular Biology and Biotechnology, FORTH, 71110 Heraklion, Greece

**Keywords:** *Laurencia*, acetogenins, anti-inflammatory activity, thuwalallene, thuwalenyne

## Abstract

Thuwalallenes A–E (**1**–**3**, **5** and **8**) and thuwalenynes A–C (**4**, **6**, **7**), new C_15_ acetogenins featuring uncommon ring systems, along with *cis*-maneonene D (**9**), thyrsiferol (**10**) and 23-acetyl-thyrsiferol (**11**) were isolated from the organic extract of a population of the red alga *Laurencia* sp., collected at Rose Reef off the village of Thuwal in the Red Sea waters of the Kingdom of Saudi Arabia. The structure elucidation of the isolated natural products was based on extensive analysis of their spectroscopic data. Compounds **1**–**6**, **8**, **10** and **11** were evaluated for their anti-inflammatory activity by quantifying nitric oxide (NO) release in response to TLR4 stimulation in macrophages. Besides compound **4** that did not exhibit any activity, all other tested metabolites inhibited NO production from activated macrophages. Among them, thyrsiferol (**10**) and 23-acetylthyrsiferol (**11**) displayed activity with IC_50_ values in the low nM scale without cytotoxicity.

## 1. Introduction

The genus *Laurencia* (Rhodomelaceae) is a cosmopolitan genus, comprising *ca.* 140 accepted species [1]. Red algae of this genus occur mainly in temperate, subtropical and tropical coastal environments, littoral to sublittoral, throughout the world, down to 65 m depth [1]. Τhe taxonomy of the genus has undergone important revisions and is still a subject of debate, due to the diversity and/or the plasticity of the markers used for the distinction of taxa. Apart from the challenging taxonomy, the genus presents a wide chemical diversity and an unparalleled ability to produce a large variety of secondary metabolites, including C_15_ acetogenins, sesquiterpenes, diterpenes and triterpenes, often with a high degree of halogenation, conferring effective chemical defence against herbivores [2,3]. This remarkable capacity of *Laurencia* to biosynthesize such a broad range of secondary metabolites could serve as an important tool in the chemotaxonomy of the genus. A number of these compounds have exhibited an array of pharmacological activities, such as anti-tumor, anti-inflammatory, antibacterial and antifungal [4]. Despite the fact that throughout the last 60 years *Laurencia* has been extensively studied, and now acknowledged as the most heavily investigated genus of red algae (Rhodophyta), new compounds are still being isolated [5,6,7]. In the context of our ongoing interest on the chemical diversity of this genus and the bioactivity potential of its secondary metabolites [2,7,8,9,10], we investigated the chemistry of a *Laurencia* sp. population collected near the village of Thuwal in the central Saudi Arabian Red Sea. Herein, we report the isolation and structure elucidation of 11 compounds (**1**–**11**), among which **1**–**8** are new natural products, and the evaluation of their anti-inflammatory activity.

## 2. Results and Discussion

### 2.1. Structure Elucidation of the Isolated Metabolites

**Figure 1:**
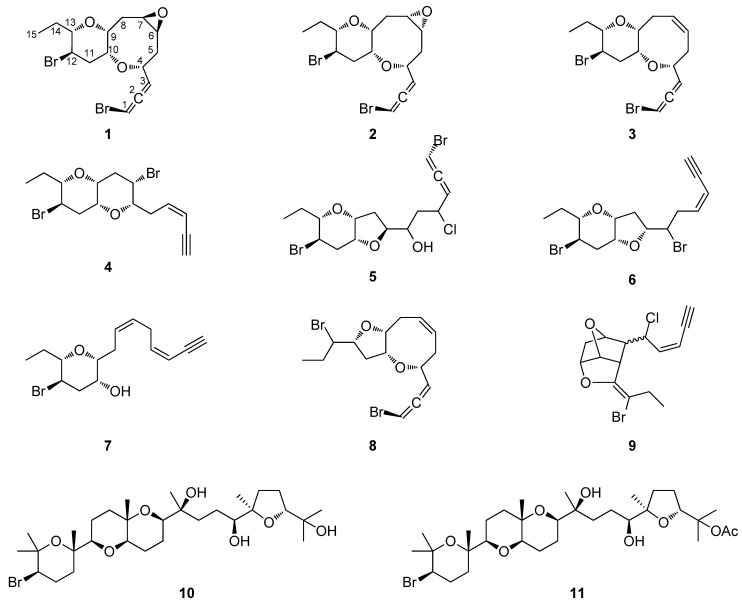
Chemical structures of compounds **1**–**11**.

The organic extract of specimens of a Saudi Arabian *Laurencia* sp. population was subjected to a series of chromatographic separations to yield 11 compounds (Figure 1), including eight new C_15_ acetogenins (**1**–**8**) and three previously reported metabolites, which were identified as *cis*-maneonene D (**9**) [11], thyrsiferol (**10**) [12] and 23-acetylthyrsiferol (**11**) [13] by comparison of their spectroscopic and physical characteristics with those reported in the literature.

**Figure 2:**
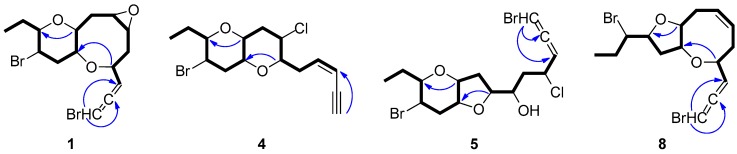
COSY and key HMBC correlations of compounds **1**, **4**, **5** and **8**.

**Figure 3:**
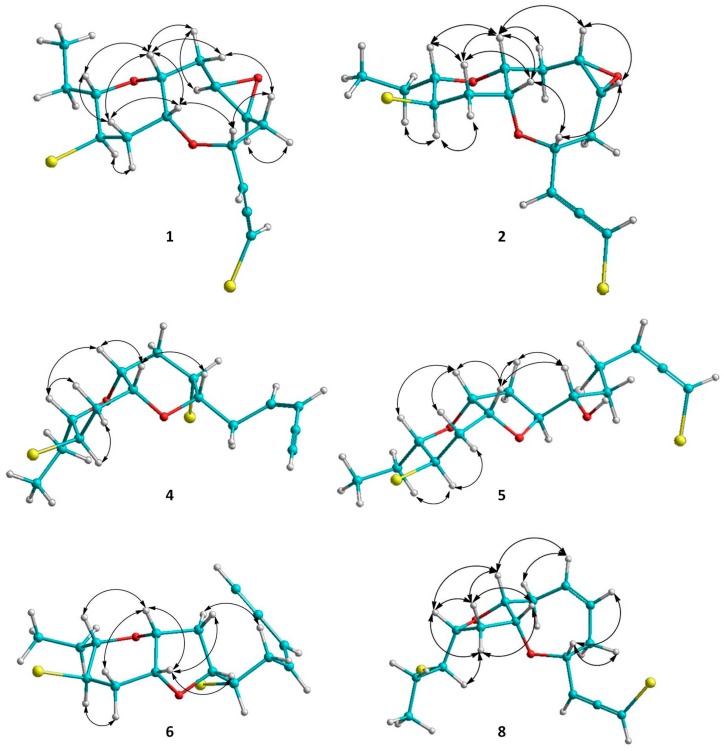
Κey ΝOΕ correlations of compounds **1**, **2**, **4**, **5**, **6** and **8**.

Thuwalallene A (**1**) was isolated as a colorless oil with the molecular formula C_15_H_20_Br_2_O_3_, as indicated by its HR-APCIMS and NMR data. The HSQC and HMBC spectra confirmed the presence of fifteen carbon atoms, corresponding to one non-protonated carbon, nine methines, four methylenes and one methyl (Table 1). A bromoallene moiety was evident from the chemical shifts of the allenic carbons at *δ*_C_ 201.5, 102.7 and 75.7, while the presence of seven deshielded methines bearing halogen or oxygen atoms at *δ*_C_ 83.7, 78.6, 76.3, 73.6, 52.7, 52.3 and 48.6 was observed. Additionally, in the ^1^H-NMR spectrum (Table 2) obvious were the signals for a methyl on a secondary carbon (*δ*_H_ 0.95) and seven methines resonating at *δ*_H_ 4.36, 4.11, 3.58, 3.36, 3.32, 3.19, and 3.09 attributed to protons of oxygenated or halogenated carbons. Since the allene moiety accounted for two of the five degrees of unsaturation, the molecular structure of **1** was determined as tricyclic. The cross-peaks observed in the COSY spectrum revealed a sole spin system extending from C-3 to C-15, placing the heteroatoms at C-4, C-6, C-7, C-9, C-10, C-12 and C-13. The HMBC correlations of H-4 to C-10 and H-9 to C-13 determined the presence of an oxocane ring and a tetrahydropyran, thus establishing the rare 4,10:9,13-bisepoxy core in the molecule (Figure 2). The third oxygen atom, in conjunction with the chemical shifts of C-6 and C-7 mandated the presence of an epoxy ring, thus completing the planar structure of metabolite **1**. The relative configuration of the stereogenic centers of **1** was proposed on the basis of the key correlations displayed in the NOESY spectrum (Figure 3) and the measured coupling constants. In particular, the coupling constants of H-12 (*J* = 12.5, 10.3, 4.0 Hz) established its axial orientation. The NOE cross-peaks of H-12 with H-11α (*δ*_H_ 2.45), of H-13 with H-9 and of the latter with H-10, as well as of H-11β (*δ*_H_ 2.04) with H-9, H-10 and H-13 determined the *cis* fusion of the tetrahydropyran and oxocane rings and established the relative configuration at C-9, C-10, C-12 and C-13. Furthermore, the NOE enhancement of H-4 with H-10 determined their *cis* orientation. Additionally, the NOE correlations of H-9 with both H-8α (*δ*_H_ 2.59) and H-8β (*δ*_H_ 1.33), of H-8α with H-7, of H-5β (*δ*_H_ 1.65) with both H-4 and H-8β and of H-5α (*δ*_H_ 2.25) with H-6 established the relative configuration at C-4, C-6, and C-7. According to the empirical rule proposed by Lowe about the absolute configuration of chiral allenes [14,15], the strong negative value of the optical rotation measured for compound **1** was indicative of the 2*R* configuration of the bromoallene moiety. Thus, the relative configurations of the asymmetric centers of **1** were established as 2*R*,4R*,6S*,7R*,9R*,10R*,12R*,13S*.

Thuwalallene B (**2**), obtained as a colorless oil, exhibited the same molecular formula as **1** according to its HR-APCIMS and NMR data. Compound **2** exhibited rather similar spectroscopic data to those of **1** (Table 1 and Table 2), suggesting that compounds **1** and **2** were stereoisomers. Indeed, characteristic correlations of a bromoallene moiety (*δ*_C_ 201.9, 102.0 and 73.7), along with signals of seven heteroatom-bearing methines (*δ*_C_ 82.7, 79.5, 76.2, 75.6, 52.4, 51.8 and 48.6), were observed for **2** in its HSQC and HMBC spectra. After thorough analysis of the homonuclear correlations observed in the COSY spectrum of **2**, the same spin system extending from C-3 to C-15 was identified, while on the basis of the heteronuclear correlations displayed in the HMBC spectrum, ether linkages between C-4 and C-10, C-9 and C-13 and C-6 and C-7 were observed, as in the case of **1**, confirming the same gross structure. The relative configuration of compound **2** was elucidated after detailed analysis of the NOE enhancements observed and the measured coupling constants (Figure 3). As in the case of **1**, the relative configurations at C-4, C-9, C-10, C-12 and C-13 were determined as 4R*,9R*,10R*,12R*,13S* on the basis of the NOE interactions of H-4 and H-10, of H-12 and both H-11α (*δ*_H_ 2.57) and H-14b (*δ*_H_ 1.49), of H-9 and both H-10 and H-13, and of H-11β (*δ*_H_ 2.08) with H-9, H-10 and H-13. In contrast, the NOE correlations of H-9 with both H-8β (*δ*_H_ 2.39) and H-7 and of H-6 with both H-4 and H-7 established the relative configuration at C-6 and C-7 as 6R*,7S*. The intense negative optical rotation measured for compound **2**, which was determined as the 6,7-stereoisomer of **1**, was again indicative of the 2*R* configuration of the bromoallene moiety.

Thuwalallene C (**3**) was obtained as a colorless oil. Its molecular formula was deduced as C_15_H_20_Br_2_O_2_ on the basis of its HR-APCIMS and NMR data. The NMR spectroscopic data of **3** (Table 1 and Table 2) showed a close resemblance to those of **1** and **2**. The main difference was the absence of the two oxygenated methines attributed to H-6 and H-7, while their replacement by two olefinic methines resonating at *δ*_H_ 5.71 and 5.82, which were assigned on the basis of the COSY cross-peaks observed, was obvious. The relative configurations of the asymmetric centers of **3** were determined mainly on the basis of the enhancements observed in its NOESY spectrum, in close resemblance to those of metabolite **2**, as 4R*,9R*,10R*,12R*,13S*. The absolute configuration of the bromoallene functionality was established as 2*R* on the basis of the intense negative value of the measured optical rotation.

Thuwalenyne A (**4**) was isolated as a colorless oil and exhibited the molecular formula C_15_H_20_Br_2_O_2_, as determined on the basis of its HR-APCIMS and NMR data (Table 1 and Table 2). The HSQC and HMBC spectra of **4** displayed correlations indicative of 15 carbons corresponding to one non-protonated carbon, nine methines, four methylenes, and one methyl. Among them, four carbons were bonded to an oxygen atom each (*δ*_C_ 70.7, 75.6, 78.3 and 82.1) and two were halogenated (*δ*_C_ 46.4 and 47.1). In addition, the chemical shift of the quaternary carbon at *δ*_C_ 79.9, along with the resonances of three tertiary carbons at *δ*_C_ 82.1, 111.1 and 140.2 were indicative of a terminal -enyne moiety. The geometry of the double bond was determined as *Z* on the basis of the coupling constant value (*J* = 10.7 Hz) measured between the olefinic methines H-3 and H-4, as also suggested by the chemical shift of the acetylenic proton H-1 (*δ* 3.11). The COSY cross-peaks readily identified the extended spin system spanning from C-3 to C-15, while the HMBC correlations of H-6 to C-10 and of H-9 to C-13 established a 2,7-dioxabicyclo[4.4.0]decane ring system (Figure 2). The relative configuration of the asymmetric centers of **4** was determined on the basis of the observed NOE enhancements and measured coupling constants (Figure 3). The strong NOE interactions of H-6/H-10, H-10/H-9, and H-9/H-13 revealed the *cis* fusion of the two pyran rings and the coplanar orientation of H-6, H-9, H-10 and H-13. Furthermore, the NOE enhancements of H-9/H-11β and of H-11α/H-12 determined the relative configuration at C-12, whereas the coupling constants measured for H-12 (*J* = 12.1, 10.0, 4.4 Hz) established its axial orientation. The fact that H-7 appeared as a broad singlet indicated its equatorial orientation, which in conjunction with its NOE interaction with H-6 established the relative configuration at C-7. Thus, the relative configurations of the chiral centers of **4** were determined as 6S*,7S*,9R*,10R*,12R*,13S*.

Thuwalallene D (**5**) was obtained as a colorless oil. The HR-APCIMS spectrum exhibited isotopic pseudomolecular ion peaks [M + H]^+^ at *m*/*z* 442.9602, 444.9579, 446.9557 and 448.9528 with a ratio of 51:100:49:15, characteristic for the presence of one chlorine and two bromine atoms in the molecule. Based on the HR-APCIMS and NMR data, the molecular formula of **5** was deduced as C_15_H_21_Br_2_ClO_3_. The structural elements of **5** included a bromoallene functionality (*δ*_C_ 200.5, 102.1, 75.1), seven halogenated or oxygenated methines (*δ*_C_ 81.5, 80.2, 78.3, 77.7, 70.6, 54.5, 47.4), four methylenes (*δ*_C_ 40.9, 38.4, 35.8, 26.1) and a methyl (*δ*_C_ 8.8). The cross-peaks observed in the COSY spectrum, in combination with the HMBC correlations of H-7 with C-10 and of H-9 with C-13 established a 7,10:9,13-bisepoxy core and placed the chlorine atom at C-4, a hydroxy group at C-6 and the second bromine atom at C-12 (Figure 2). The strong NOE enhancement between H-9 and H-10 suggested the *cis* fusion of the two rings. Moreover, the coupling constants of H-12 (*J* = 11.8, 10.2, 4.3 Hz) established its axial orientation, while the NOE interactions of H-9/H-13, H-11α/H-12, H-11β/H-9 and H-12/H-14b (*δ*_H_ 1.52) defined the relative configurations of the chiral centers C-9, C-10, C-12 and C-13 of the pyran ring. The NOE cross-peaks of H-8β with both H-6 and H-10, as well as the weak, albeit observable, NOE correlation of H-6 with H-10 suggested the relative configuration at C-7. The relative configuration at C-4 and C-6 could not be safely assigned based solely on NOE data. On this basis, the relative configurations of the chiral centers of **5** were determined as 7S*,9R*,10R*,12R*,13S* (Figure 3), while the strong negative value of the optical rotation measured indicated the 2*R* configuration of the bromoallene.

Thuwalenyne B (**6**) was obtained as a colorless oil. Its molecular formula was deduced as C_15_H_20_Br_2_O_2_ on the basis of HR-APCIMS and NMR data (Table 1 and Table 2). As in the case of **5**, analysis of the correlations displayed in COSY, HSQC and HMBC spectra of **6** established the same 7,10:9,13-bisepoxy core, with the main difference being the presence of an -enyne terminus, evident from the chemical shift of the quaternary carbon at *δ*_C_ 81.8, along with the resonances of three tertiary carbons at *δ*_C_ 141.6, 110.5 and 80.0, instead of a bromoallene moiety. The geometry of the 1,2-disubstituted double bond was defined as *Z* on the basis of the coupling constant (*J* = 10.7 Hz) measured between the olefinic H-3 and H-4, a fact which was corroborated by the chemical shift of the acetylenic proton H-1 (*δ* 3.11). The relative configuration of **6** was elucidated after thorough analysis of the NOE enhancements and the measured coupling constants (Figure 3). The NOE interaction between H-9 and H-10 determined the *cis* fusion of the tetrahydropyran and tetrahydrofuran rings. The axial orientation of H-12 was determined on the basis the observed coupling constants (*J* = 11.8, 10.2, 4.5 Hz), while the NOE interactions of H-9/H-13, H-9/H-11β and H-11α/H-12 established the coplanar orientation of H-9 and H-13. The NOE enhancements of H-10 with both H-7 and H-8β, as well as of H-5a (*δ*_H_ 3.02) with H-8α determined the relative configuration at C-7, while the relative configuration at C-6 could not be safely assigned based solely on NOE data. Thus, the relative configurations of the asymmetric centers of **6** were assigned as 7R*,9R*,10R*,12R*,13S*.

Thuwalenyne C (**7**)**,** obtained as a colorless oil, possessed the molecular formula C_15_H_21_BrO_2_, as determined by the HR-APCIMS and NMR data (Table 1 and Table 2). The presence of an -enyne functionality, as observed from the ^1^H and ^13^C chemical shifts, along with the isolated double bond accounted for four of the five degrees of unsaturation, thus indicating that **7** was a monocyclic C_15_ acetogenin. The HMBC correlation from H-9 to C-13 led to the identification of one tetrahydropyran ring in the structure of **7**, while the COSY cross-peaks placed the isolated double bond between C-6 and C-7, as well as a hydroxy group at C-10 and the bromine atom at C-12. Thorough analysis of the NOE enhancements in combination with the observed coupling constants led to the determination of the relative configurations of the chiral centers of **7**. The coupling constant of H-12/H-13 (*J* = 9.9 Hz) indicated their diaxial orientation, whereas the NOE interactions of H-9/H-13, H-11α/H-12 and H-9/H-11β established the relative configuration at C-9, C-12 and C-13. The equatorial orientation of H-10, and thus the relative configuration at C-10, was established based on the small coupling constants of H-10 (brs). The geometry of the 1,2 disubstituted double bond of the –enyne moiety was determined as *Z* according to the coupling constant (*J* = 10.9 Hz) measured between H-3 and H-4 and the chemical shift of the acetylenic proton resonating at *δ*_H_ 3.10. Furthermore, the geometry of the Δ^6^ double bond was assigned as *Z* on the basis of the resonance of the doubly allylic methylene carbon C-5 at *δ*_c_ 28.0 [16], as well as the NOE cross-peak of H_2_-5/H_2_-8. Thus, the relative configurations of the asymmetric centers of **7** were established as 9R*,10R*,12R*,13S*.

Thuwalallene E (**8**)**,** isolated as a colorless oil, displayed the molecular formula C_15_H_20_Br_2_O_2_, as indicated from the HR-APCIMS and NMR data. The NMR spectroscopic features of **8** resembled those of **3** (Table 1 and Table 2). A single spin system spanning from C-3 to C-15 was identified based on the COSY correlations. As in the case of **3**, the HMBC correlation from H-4 to C-10 identified an oxocane ring. However, the HMBC correlation from H-9 to C-12 instead to C-13 established a tetrahydrofuran instead of a tetrahydropyran as the second ring of the bicyclic system of **8** (Figure 2). The NOE correlations of H-4/H-5β, H-4/H-6, H-7/H-8β, H-7/H-9, H-9/H-11β, H-9/H-12, H-10/H-11β and H-11β/H-12, as well as of H-10/H-11α, H-11α/H-12 and H-11α/H-13 established the *cis* fusion of the 4,10:9,12-bisepoxy ring system and the coplanar orientation of H-4, H-9, H-10 and H-12, thus determining the relative configurations at C-4, C-9, C-10, C-12 as 4R*,9R*,10R*,12R* (Figure 3). Furthermore, the 2*R* configuration of the bromoallene was assigned on the basis of the intense negative value of its measured optical rotation.

### 2.2. Evaluation of the Anti-inflammatory Activity of the Isolated Metabolites

Compounds **1**–**6**, **8**, **10** and **11** were evaluated for their anti-inflammatory activity using the Griess reaction to quantify the release of nitric oxide (NO), a major pro-inflammatory mediator, in response to TLR4 stimulation in macrophages, whereas the bioactivity of metabolites **7** and **9** was not evaluated since they were proven to be unstable. The IC_50_ values for the inhibition of NO production were determined in LPS-treated RAW 264.7 cells in comparison to cells treated with the vehicle (Carbowax 400) only (Table 3, Figure S1). To verify that the anti-inflammatory activity observed was not due to cytotoxic effect, the potential cytotoxicity of **1**–**6**, **8**, **10** and **11** was evaluated using the MTT assay following 24, 48 and 72 h of treatment in RAW 264.7 macrophages (Table 3, Figure S2).

Compounds **1** and **2** exhibited IC_50_ values of 26.03 μΜ and 13.23 μΜ, with cytotoxicity at concentrations higher than 15.62 μΜ and 31.25 μΜ, respectively, 72 h following treatment. Compound **3**, although displaying structural similarity to **1** and **2**, showed a lower IC_50_ value (4.18 μΜ) and cytotoxicity at concentrations higher than 15.62 μΜ at 72 h of treatment. Compound **4** did not exhibit anti-inflammatory activity or cytotoxicity at the concentrations tested. Compound **6** exhibited weak anti-inflammatory activity with an IC_50_ value of 37.39 μΜ, which was primarily attributed to its cytotoxic activity since it exhibited toxicity to RAW 264.7 macrophages at concentrations higher than 15.62 μΜ. On the contrary, **5** displayed anti-inflammatory activity with an IC_50_ value of 12.41 μΜ but significant cytotoxicity only at concentrations above 15.62 μΜ at 72 h of treatment. Interestingly, **8** exhibited potent anti-inflammatory activity with an IC_50_ value of 3.98 μΜ, whereas it was not cytotoxic at concentrations below 7.81 μΜ, 72 h following treatment. The most potent anti-inflammatory activity was observed for **10** and **11** which exhibited IC_50_ values of 4.387 nM and 2.633 nM, respectively. Compound **10** exhibited significant cytotoxicity at concentrations above 100 nM, whereas **11** at concentrations above 10 nM, indicating that the potent anti-inflammatory activity of both **10** and **11** was not due to their cytotoxicity. To the best of our knowledge, this is the first report on the anti-inflammatory activity of C_15_ acetogenins.

## 3. Materials and Methods

### 3.1. General Experimental Procedures

Optical rotations were measured on a Krüss polarimeter (A. KRÜSS Optronic GmbH, Hamburg, Germany) equipped with a 0.5 dm cell. UV spectra were recorded on a Lambda 40 UV/Vis spectrophotometer (Perkin Elmer Ltd., Beaconsfield, UK). IR spectra were obtained on a Alpha II FTIR spectrometer (Bruker Optik GmbH, Ettlingen, Germany). High-resolution APCI mass spectra were measured on a LTQ Orbitrap Velos mass spectrometer (Thermo Fisher Scientific, Bremen, Germany). NMR spectra were recorded on Avance NEO 950, Avance NEO 700, Avance III 600, and DRX 400 spectrometers (Bruker BioSpin GmbH, Rheinstetten, Germany). Chemical shifts are given on a *δ* (ppm) scale using TMS as internal standard. The 2D experiments (HSQC, HMBC, COSY, NOESY) were performed using standard Bruker pulse sequences. Column chromatography separations were performed with Kieselgel 60 (Merck, Darmstadt, Germany). HPLC separations were conducted using a Waters 600 liquid chromatography pump equipped with a Waters 410 differential refractometer (Waters Corporation, Milford, MA, USA), using a 25 cm × 10 mm Econosphere Silica 10 μ column (Grace, Columbia, MD, USA). TLC were performed with Kieselgel 60 F_254_ aluminum plates (Merck, Darmstadt, Germany) and spots were detected after spraying with 20% H_2_SO_4_ in MeOH reagent and heating at 100 °C for 1 min.

### 3.2. Biological Material

Specimens of *Laurencia* sp. were collected by hand from Rose Reef (22°18′ N, 38°53′ E) off the village of Thuwal in the Red Sea coast of the Kingdom of Saudi Arabia, at a depth of 1.5–2 m in January 2018. A voucher specimen of the alga has been deposited at the Herbarium of the Section of Pharmacognosy and Chemistry of Natural Products, Department of Pharmacy, National and Kapodistrian University of Athens (ATPH/MP0677).

### 3.3. Extraction and Isolation

The fresh algal specimens were exhaustively extracted with mixtures of CH_2_Cl_2_/MeOH (1:1) at room temperature. After evaporation of the solvent in vacuo, the organic extract (288.0 mg) was subjected to vacuum liquid chromatography over silica gel using cHex with increasing amounts of EtOAc and finally MeOH as the mobile phase to yield 7 fractions (A–G), among which **10** (11.7 mg) and **11** (8.0 mg) were isolated in pure form. Fraction A (102.8 mg) was subjected to vacuum liquid chromatography over silica gel using mixtures of *n*Hex and EtOAc of increasing polarity as eluent to afford 7 fractions (A1–A7). Fraction A2 (37.9 mg) was repeatedly purified by normal-phase HPLC using cHex/EtOAc (98:2) as the mobile phase to afford **3** (2.9 mg), **4** (1.5 mg), **6** (4.1 mg) and **8** (1.4 mg). Fraction A5 (6.7 mg) was purified by normal-phase HPLC using cHex/EtOAc (95:5) and subsequently *n*Hex/EtOAc (94:6) as the mobile phase to yield **9** (1.5 mg). Fraction A6 (4.0 mg) was subjected to normal-phase HPLC using cHex/EtOAc (83:17) as the mobile phase to yield **7** (2.0 mg). Fraction B (65.6 mg) was subjected to normal-phase HPLC using cHex/EtOAc (83:17) and subsequently cHex/acetone (95:15) as the mobile phase to afford **1** (33.0 mg), **2** (3.3 mg), **5** (3.8 mg) and **7** (2.2 mg).

*Thuwalallene A* (**1**): Colorless oil; [α]${}_{D}^{20}$ −158 (*c* 1.0, CHCl_3_); UV (CHCl_3_) *λ*_max_ (log *ε*) 241 (3.30); IR (thin film) *ν*_max_ 2961, 2925, 2880, 2855, 1723, 1480, 1439, 1090, 660 cm^−1^; ^1^H and ^13^C NMR data, see Table 1; Table 2; HR-APCIMS *m/z* 406.9839, 408.9818, 410.9797 [M + H]^+^ (54:100:48) (calcd. for C_15_H_21_^79^Br_2_O_3_, 406.9852, C_15_H_21_^79^Br^81^BrO_3_, 408.9831, C_15_H_21_^81^Br_2_O_3_, 410.9811).

*Thuwalallene B* (**2**): Colorless oil; [α]${}_{D}^{20}$ −77 (*c* 0.30, CHCl_3_); UV (CHCl_3_) *λ*_max_ (log *ε*) 242 (2.98); IR (thin film) *ν*_max_ 2961, 2927, 2876, 2853, 1713, 1452, 1382, 1085, 779, 662 cm^−1^; ^1^H and ^13^C NMR data, see Table 1; Table 2; HR-APCIMS *m/z* 406.9846, 408.9825, 410.9804 [M + H]^+^ (54:100:50) (calcd. for C_15_H_21_^79^Br_2_O_3_, 406.9852, C_15_H_21_^79^Br^81^BrO_3_, 408.9831, C_15_H_21_^81^Br_2_O_3_, 410.9811).

*Thuwalallene C* (**3**): Colorless oil; [α]${}_{D}^{20}$ −49 (*c* 0.25, CHCl_3_); UV (CHCl_3_) *λ*_max_ (log *ε*) 242 (2.97); IR (thin film) *ν*_max_ 2961, 2927, 2851, 1080, 764, 658 cm^−1^; ^1^H and ^13^C NMR data, see Table 1; Table 2; HR-APCIMS *m/z* 390.9890, 392.9868, 394.9847 [M + H]^+^ (53:100:50) (calcd. for C_15_H_21_^79^Br_2_O_2_, 390.9903, C_15_H_21_^79^Br^81^BrO_2_, 392.9882, C_15_H_21_^81^Br_2_O_2_, 394.9862).

*Thuwalenyne A* (**4**): Colorless oil; [α]${}_{D}^{20}$ +9 (*c* 0.10, CHCl_3_); UV (CHCl_3_) *λ*_max_ (log *ε*) 242 (3.14); IR (thin film) *ν*_max_ 3292, 2959, 2925, 2855, 1112, 1093, 1059, 998 cm^−1^; ^1^H and ^13^C NMR data, see Table 1; Table 2; HR-APCIMS *m/z* 390.9889, 392.9865, 394.9843 [M + H]^+^ (50:100:50) (calcd. for C_15_H_21_^79^Br_2_O_2_, 390.9903, C_15_H_21_^79^Br^81^BrO_2_, 392.9882, C_15_H_21_^81^Br_2_O_2_, 394.9862).

*Thuwalallene D* (**5**): Colorless oil; [α]${}_{D}^{20}$ −62 (*c* 0.33, CHCl_3_); UV (CHCl_3_) *λ*_max_ (log *ε*) 241 (3.08); IR (thin film) *ν*_max_ 3434, 2925, 2857, 1730, 1108, 1059, 660 cm^−1^; ^1^H and ^13^C NMR data, see Table 1; Table 2; HR-APCIMS *m/z* 442.9602, 444.9579, 446.9557 and 448.9528 [M + H]^+^ (44:100:67:15) (calcd. for C_15_H_22_^79^Br_2_^35^ClO_3_, 442.9619, C_15_H_22_^79^Br^81^Br^35^ClO_3_, C_15_H_22_^79^Br_2_^37^ClO_3_, 444.9518, C_15_H_22_^81^Br_2_^35^ClO_3_, C_15_H_22_^79^Br^81^Br^37^ClO_3_, 446.9578, C_15_H_22_^81^Br_2_^37^ClO_3_, 448.9548).

*Thuwalenyne B* (**6**): Colorless oil; [α]${}_{D}^{20}$ −7 (*c* 0.36, CHCl_3_); UV (CHCl_3_) *λ*_max_ (log *ε*) 242 (3.08); IR (thin film) *ν*_max_ 3300, 2959, 2925, 2882, 2857, 1437, 1108, 1100, 1057, 616 cm^−1^; ^1^H and ^13^C NMR data, see Table 1; Table 2; HR-APCIMS *m/z* 390.9891, 392.9868, 394.9846 [M + H]^+^ (52:100:48) (calcd. for C_15_H_21_^79^Br_2_O_2_, 390.9903, C_15_H_21_^79^Br^81^BrO_2_, 392.9882, C_15_H_21_^81^Br_2_O_2_, 394.9862).

*Thuwalenyne C* (**7**): Colorless oil; [α]${}_{D}^{20}$ −15 (*c* 0.25, CHCl_3_); UV (CHCl_3_) *λ*_max_ (log *ε*) 240 (3.61); IR (thin film) *ν*_max_ 3287, 2926, 1717, 1076; ^1^H and ^13^C NMR data, see Table 1; Table 2; HR-APCIMS *m/z* 311.0631, 313.0609 [M − H]^−^ (100:98) (calcd. for C_15_H_20_^79^BrO_2_, 311.0652, C_15_H_20_^81^BrO_2_, 313.0632).

*Thuwalallene E* (**8**): Colorless oil; [α]${}_{D}^{20}$ −38 (*c* 0.10, CHCl_3_); UV (CHCl_3_) *λ*_max_ (log *ε*) 243 (2.84); IR (thin film) *ν*_max_ 2967, 2933, 2880, 2853, 1711, 1063, 800, 660 cm^−1^; ^1^H and ^13^C NMR data, see Table 1; Table 2; HR-APCIMS *m/z* 390.9896, 392.9877, 394.9857 [M + H]^+^ (53:100:47) (calcd. for C_15_H_21_^79^Br_2_O_2_, 390.9903, C_15_H_21_^79^Br^81^BrO_2_, 392.9882, C_15_H_21_^81^Br_2_O_2_, 394.9862).

### 3.4. Cell Culture

Mouse macrophage cell line RAW 264.7 was cultured in DMEM medium (cat. # 21885-025, Gibco, Thermo Fisher Scientific, Waltham, MA, USA) supplemented with 10% heat inactivated fetal bovine serum (cat. # 10270-106, Gibco, Thermo Fisher Scientific, Waltham, MA, USA) and 1% penicillin-streptomycin (cat. # 15070-063, Gibco, Thermo Fisher Scientific, Waltham, MA, USA). Cells were cultured in 37 °C and 5% CO_2_. Each compound was diluted in Carbowax^TM^ 400 + 10% ethanol (cat. # 1.00983, Merck, Darmstadt, Germany), used also as control solvent. Final concentration in culture was 0.1% v/v carbowax and 0.01% v/v ethanol. RAW 264.7 macrophages were activated using 100 ng/mL lipopolysaccharide (LPS) (L2630, Merck, Darmstadt, Germany). In IC_50_ determination experiments, macrophages were pre-treated for 1 h with the respective compound prior to LPS stimulation.

### 3.5. Nitric Oxide Measurement

30 × 10^4^ RAW 264.7 mouse macrophages were plated in 24-well plates over-night with 0.5 mL complete medium. Cells were pretreated for 1 h with the respected compound concentration and then stimulated with 100 ng/mL LPS (L2630, Merck, Darmstadt, Germany) for 48 h. The amount of nitrite, an oxidative product of NO, was measured in culture supernatant of each sample using the Griess reaction. 100 μL of supernatant was mixed with 100 μL of sulfanilamide solution (1% sulfanilamide in 5% H_3_PO_4_) and incubated for 5 min at room temperature. Then, 100 μL of NED solution (0.1% N-1-napthylethylenediamine dihydrochlorite in H_2_O) was added and the absorbance was measured in an automated microplate reader (Infinite 200 PRO, Tecan, Männedorf, Switzerland) at 540 nm. Nitrite concentration was calculated using a sodium nitrite standard curve. All incubations were performed in the dark.

### 3.6. MTT Measurement

3.5 × 10^3^ RAW 264.7 mouse macrophages were seeded in 96-well plate and cultured over night. Cells were subsequently treated with the respective compound concentration and incubated for 24, 48 and 72 h. Number of cells was measured prior to treatment and used as normalisation control. Thiazolyl Blue Tetrazolium Bromide (MTT) (A2231.001, Applichem GmbH, Darmstadt, Germany) was added to the cells in a final concentration of 0.5 mg/mL and then cells were incubated at 37 °C and 5% CO_2_ for 4 h. The supernatant was discarded and cells were lysed with 2-propanol (33539, Honeywell, Charlotte, NC, USA) with 0.4% HCl (30721, Merck, Darmstadt, Germany). The absorbance of each sample was measured in an automated microplate reader (Infinite 200 PRO, Tecan, Männedorf, Switzerland) at 600 nm. The average OD of each treated sample was normalized to the OD of the control sample and statistical analysis was performed using Graphpad Prism 7.0 (GraphPad Software, San Diego, CA, USA).

### 3.7. Statistical Analysis

All data are presented as mean ± SEM and as percentage in case of IC_50_ evaluation. Statistical analysis was performed using Graphpad Prism 7.0 (GraphPad Software, San Diego, CA, USA). D’Agostino & Pearson, Shapiro Wilk and KS tests were used to evaluate normality. In case of normality, one-way ANOVA was performed, whereas in all other cases the non-parametric Kruskal-Wallis test was used. Differences with a *p* value < 0.05 are considered significant (* indicates *p* < 0.05, ** indicates *p* < 0.01, *** indicates *p* < 0.001).

## 4. Conclusions

The chemical investigation of the organic extract of a population of the red alga *Laurencia* sp., collected at Rose Reef off the village of Thuwal in the Kingdom of Saudi Arabia, resulted in the isolation and structure elucidation of thuwalallenes A–E (**1**–**3**, **5** and **8**) and thuwalenynes A–C (**4**, **6**, **7**), new C_15_ acetogenins, along with *cis*-maneonene D (**9**), thyrsiferol (**10**) and 23-acetylthyrsiferol (**11**). Among the new metabolites, **1**–**6** and **8** feature uncommon or unprecedented bicyclic ring systems. Specifically, **1**–**3** possess a 4,10:9,13-bisepoxy core, **4** has a 2,7-dioxabicyclo[4.4.0]decane system, **5** and **6** feature a 7,10:9,13-bisepoxy core, while **8** incorporates a 4,10:9,12-bisepoxy system that is reported for the first time. Compounds **1**–**6**, **8**, **10** and **11** were evaluated for their anti-inflammatory activity by quantifying NO release in response to TLR4 stimulation in macrophages. All tested metabolites, except compound **4**, inhibited NO production, with the triterpenes thyrsiferol (**10**) and 23-acetyl-thyrsiferol (**11**) displaying activity with IC_50_ values in the low nM scale without significant cytotoxicity.

## Figures and Tables

**Table 1:** ^13^C-NMR data (*δ* in ppm) in CDCl_3_ of compounds **1**–**8**.

Position	1 ^1,2^	2 ^1,3^	3 ^4^	4 ^1,3^	5 ^1,3^	6 ^1,5^	7 ^1,5^	8 ^4^
1	75.7	73.7	73.8	82.1	75.1	80.0	80.7	74.2
2	201.5	201.9	201.0	79.9	200.5	81.8	78.6	200.9
3	102.7	102.0	103.2	111.1	102.1	110.5	108.0	103.8
4	73.6	75.6	78.0	140.2	54.5	141.6	142.9	79.9
5	31.2	36.3	34.2	35.6	40.9	34.2	28.0	34.5
6	52.3	52.4	129.6	78.3	70.6	55.6	127.9	128.9
7	52.7	51.8	129.4	46.4	81.5	80.9	126.0	130.1
8	32.6	29.9	30.1	36.5	35.8	36.1	29.1	28.8
9	76.3	76.2	79.7	70.7	77.7	76.1	79.7	85.9
10	78.6	79.5	77.7	75.6	78.3	79.4	69.1	83.1
11	43.5	42.1	42.7	40.5	38.4	37.7	43.0	39.7
12	48.6	48.6	49.3	47.1	47.4	47.4	47.6	81.7
13	83.7	82.7	83.2	82.1	80.2	80.6	83.5	61.9
14	26.0	26.0	26.4	25.7	26.1	26.0	25.5	28.1
15	9.2	9.3	9.6	7.9	8.8	8.8	9.1	11.6

^1^ Chemical shifts were determined through HMBC correlations. ^2^ Recorded at 100 MHz. ^3^ Recorded at 150 MHz. ^4^ Recorded at 175 MHz. ^5^ Recorded at 237.5 MHz.

**Table 2:** ^1^H-NMR data (*δ* in ppm, *J* in Hz) in CDCl_3_ of compounds **1**–**8**.

Position	1 ^1^	2 ^2^	3 ^3^	4 ^2^	5 ^2^	6 ^4^	7 ^4^	8 ^3^
1	6.11 dd (5.6, 2.6)	6.06 dd (5.5, 2.1)	6.02 dd (5.6, 1.8)	3.11 brs	6.13 dd (5.7, 1.7)	3.11 d (1.6)	3.10 m	6.06 dd (5.7, 2.5)
3	5.56 dd (5.6, 4.5)	5.52 dd (5.5, 5.5)	5.52 dd (5.6, 5.6)	5.55 br d (10.7)	5.58 dd (6.9, 5.7)	5.60 br dd (10.7, 1.6)	5.46 m	5.45 dd (5.7, 4.5)
4	4.36 m	4.18 ddd (11.0, 5.5, 2.1)	4.01 m	6.07 ddd (10.7, 7.3, 7.3)	4.82 m	6.15 ddd (10.7, 7.0, 7.0)	5.93 ddd (10.9, 7.4, 7.4)	3.90 m
5	α 2.25 dd (14.3, 4.5)	α 1.67 ddd (14.6, 11.0, 9.5)	α 2.56 m	a 2.61 m	a 1.99 m	a 3.02 dddd (16.0, 7.0, 2.9, 1.6)	3.10 m	α 2.52 ddd (15.1, 10.4, 6.0)
	β 1.65 m	β 2.36 m	β 2.16 brdd (14.2, 8.1)	b 2.73 ddd (14.4, 7.3, 7.3)	b 1.74 ddd (13.4, 11.0, 1.7)	b 2.80 ddd (16.0, 7.0, 7.0)		β 2.16 m
6	3.09 ddd (10.8, 4.5, 4.5)	3.00 m	5.82 m	3.30 m	3.73 ddd (11.0, 6.1, 2.1)	4.13 m	5.48 m	5.76 m
7	3.19 ddd (9.3, 4.5, 4.5)	2.87 ddd (11.4, 3.7, 3.7)	5.71 m	4.02 brs	4.11 m	4.16 m	5.47 m	5.79 m
8	α 2.59 ddd (14.6, 5.2, 4.5)	α 1.55 m	α 2.59 m	α 2.53 brd (15.7)	α 2.05 brdd (13.3, 6.2)	α 1.99 dd (14.4, 4.3)	2.35 m	α 2.63 m
	β 1.33 ddd (14.6, 9.3, 1.6)	β 2.39 m	β 2.23 m	β 2.31 ddd (15.7, 4.3, 4.3)	β 1.82 ddd (13.3, 9.6, 3.9)	β 2.23 ddd (14.4, 9.1, 5.2)		β 2.24 m
9	3.58 brd (5.2)	3.70 ddd (11.0, 5.3, 1.1)	3.53 brdd (11.0, 5.3)	3.59 brs	4.12 m	4.06 dd (5.2, 1.7)	3.45 ddd (7.3, 7.3, 0.6)	3.84 ddd (11.3, 7.7, 3.8)
10	3.36 m	3.55 brs	3.63 brs	3.48 brs	3.91 brs	3.77 m	3.68 brs	3.89 m
11	α 2.47 ddd (12.7, 4.2, 4.2)	α 2.57 ddd (12.9, 3.6, 3.6)	α 2.52 ddd (13.3, 3.5, 3.5)	α 2.62 m	α 2.70 ddd (14.5, 4.3, 2.4)	α 2.75 ddd (14.6, 4.5, 2.3)	α 2.57 ddd (13.7, 4.5, 3.6)	α 2.10 m
	β 2.04 ddd (12.7, 12.7, 2.8)	β 2.08 ddd (12.9, 12.9, 3.6)	β 1.99 ddd (13.3, 13.3, 3.2)	β 2.08 m	β 2.13 ddd (14.5, 11.8, 3.5)	β 2.16 ddd (14.6,11.8, 3.7)	β 2.06 m	β 2.35 ddd (14.8, 8.9, 6.4)
12	4.11 ddd (12.7, 10.3, 4.2)	4.01 ddd (12.9, 10.0, 3.6)	4.02 m	4.16 ddd (12.1, 10.0, 4.4)	3.96 ddd (11.8, 10.2, 4.3)	4.04 ddd (11.8, 10.2, 4.5)	3.98 ddd (12.3, 9.9, 4.5)	4.00 ddd (8.9, 8.9, 4.5)
13	3.32 ddd (10.3, 7.9, 2.5)	3.28 ddd (10.0, 10.0, 2.3)	3.25 ddd (9.3, 9.3, 2.0)	3.33 ddd (10.0, 6.0, 2.8)	3.28 ddd (10.2, 7.8, 2.4)	3.29 ddd (10.2, 7.4, 2.6)	3.33 ddd (9.9, 9.9, 2.1)	4.05 ddd (8.9, 8.9, 2.6)
14	a 1.98 dqd (14.8, 7.3, 2.5)	a 2.01 dqd (15.2, 7.4, 2.3)	a 2.01 dqd (14.9, 7.3, 2.0)	a 1.87 dqd (14.3, 7.3, 2.8)	a 1.97 m	a 1.94 dqd (14.7, 7.4, 2.6)	a 2.04 m	a 2.16 m
	b 1.58 m	b 1.49 m	b 1.50 m	b 1.76 m	b 1.52 m	b 1.57 dqd (14.7, 7.4, 7.4)	b 1.49 m	b 1.73 m
15	0.95 t (7.3)	0.93 t (7.4)	0.96 t (7.3)	0.98 t (7.3)	0.93 t (7.4)	0.92 t (7.4)	0.96 t (7.4)	1.06 t (7.3)

^1^ Recorded at 400 MHz. ^2^ Recorded at 600 MHz. ^3^ Recorded at 700 MHz. ^4^ Recorded at 950 MHz.

**Table 3:** IC_50_ values (in μM) for inhibition of NO production and cytotoxicity of compounds **1**–**6**, **8**, **10** and **11**.

Compound	Inhibition of NO Production	Cytotoxicity (at 72 h)
**1**	26.03 ± 3.73	>15.62
**2**	13.23 ± 0.57	>31.25
**3**	4.18 ± 0.48	>15.62
**4**	>62.5	>62.50
**5**	12.41 ± 1.04	>15.62
**6**	37.39 ± 2.51	>15.62
**8**	3.98 ± 0.60	>7.81
**10**	4.387 × 10^−3^ ± 0.851 × 10^−3^	>0.10
**11**	2.633 × 10^−3^ ± 0.238 × 10^−3^	>0.01

## Supplementary Materials

The following are available online at https://www.mdpi.com/1660-3397/17/11/644/s1, Figures S1 and S2: Visual representation of the evaluation of the anti-inflammatory activity and cytotoxicity of the tested metabolites, Figures S3–S55: 1D and 2D NMR and HR-MS spectra of compounds **1**–**11**.
