# Supplementary material for: Thuwalallenes A–E and Thuwalenynes A–C: New C15 Acetogenins with Anti-Inflammatory Activity from a Saudi Arabian Red Sea Laurencia sp
Source: Mar Drugs. 2019 Nov 15;17(11):644. doi: 10.3390/md17110644 (PMC6891555; doi:10.3390/md17110644)
Supplement: Supplementary file 1 [file marinedrugs-17-00644-s001.pdf]

Supplementary Materials for

Thuwalallenes A–E and Thuwalenynes A–C: New C_{15} Acetogenins with Anti-Inflammatory Activity from a Saudi Arabian Red Sea *Laurencia* sp.

Aikaterini Koutsaviti [1], Maria G. Daskalaki [2], Susana R. Agusti [3], Sotirios C. Kampranis [2,4], Christos Tsatsanis [2,5], Carlos M. Duarte [3], Vassilios Roussis [1] and Efstathia Ioannou [1,*]

[1] Section of Pharmacognosy and Chemistry of Natural Products, Department of Pharmacy, National and Kapodistrian University of Athens, Panepistimiopolis Zografou, Athens 15771, Greece; kkoutsaviti@pharm.uoa.gr (A.K.); roussis@pharm.uoa.gr (V.R.)

[2] Laboratory of Clinical Chemistry, School of Medicine, University of Crete, Heraklion 70013, Greece; m.daskalaki@med.uoc.gr (M.G.D.); soka@plen.ku.dk (S.C.K.); tsatsani@uoc.gr (C.T.)

[3] Red Sea Research Center, King Abdullah University of Science and Technology, Thuwal, 23955-6900, Saudi Arabia; susana.agusti@kaust.edu.sa (S.R.A.); carlos.duarte@kaust.edu.sa (C.M.D.)

[4] Present address: Section of Plant Biochemistry, Department of Plant and Environmental Sciences, University of Copenhagen, Thorvaldsensvej 40, 1871 Frederiksberg C, Denmark

[5] Institute of Molecular Biology and Biotechnology, FORTH, Heraklion 71110, Greece

* Correspondence: eioannou@pharm.uoa.gr (E.I.); Tel.: +30 210 727 4913

Table of Contents

Figure S1. (**A-C**) Determination of concentration inducing 50% inhibition of NO production using LPS-treated RAW 264.7 for compounds **1–3**, **5**, **6**, **8**, **10** and **11**. S4
Figure S2. (**A-I**) Evaluation of the cytotoxic activity of compounds **1–6**, **8**, **10** and **11** by measuring proliferation rate of RAW 264.7 cells. S5
Figure S3. ^{1}H NMR spectrum of thuwalallene A (**1**) in $CDCl_3$. S6
Figure S4. HSQC spectrum of thuwalallene A (**1**) in $CDCl_3$. S6
Figure S5. HMBC spectrum of thuwalallene A (**1**) in $CDCl_3$. S7
Figure S6. COSY spectrum of thuwalallene A (**1**) in $CDCl_3$. S7
Figure S7. NOESY spectrum of thuwalallene A (**1**) in $CDCl_3$. S8
Figure S8. HR-APCIMS spectrum of thuwalallene A (**1**). S8
Figure S9. ^{1}H NMR spectrum of thuwalallene B (**2**) in $CDCl_3$. S9
Figure S10. HSQC spectrum of thuwalallene B (**2**) in $CDCl_3$. S9
Figure S11. HMBC spectrum of thuwalallene B (**2**) in $CDCl_3$. S10
Figure S12. COSY spectrum of thuwalallene B (**2**) in $CDCl_3$. S10
Figure S13. NOESY spectrum of thuwalallene B (**2**) in $CDCl_3$. S11
Figure S14. HR-APCIMS spectrum of thuwalallene B (**2**). S11
Figure S15. ^{1}H NMR spectrum of thuwalallene C (**3**) in $CDCl_3$. S12
Figure S16. ^{13}C NMR spectrum of thuwalallene C (**3**) in $CDCl_3$. S12
Figure S17. HSQC spectrum of thuwalallene C (**3**) in $CDCl_3$. S13
Figure S18. HMBC spectrum of thuwalallene C (**3**) in $CDCl_3$. S13
Figure S19. COSY spectrum of thuwalallene C (**3**) in $CDCl_3$. S14
Figure S20. NOESY spectrum of thuwalallene C (**3**) in $CDCl_3$. S14
Figure S21. HR-APCIMS spectrum of thuwalallene C (**3**). S15
Figure S22. ^{1}H NMR spectrum of thuwalenyne A (**4**) in $CDCl_3$. S16
Figure S23. HSQC spectrum of thuwalenyne A (**4**) in $CDCl_3$. S16
Figure S24. HMBC spectrum of thuwalenyne A (**4**) in $CDCl_3$. S17
Figure S25. COSY spectrum of thuwalenyne A (**4**) in $CDCl_3$. S17
Figure S26. NOESY spectrum of thuwalenyne A (**4**) in $CDCl_3$. S18
Figure S27. HR-APCIMS spectrum of thuwalenyne A (**4**). S18
Figure S28. ^{1}H NMR spectrum of thuwalallene D (**5**) in $CDCl_3$. S19
Figure S29. HSQC spectrum of thuwalallene D (**5**) in $CDCl_3$. S19
Figure S30. HMBC spectrum of thuwalallene D (**5**) in $CDCl_3$. S20
Figure S31. COSY spectrum of thuwalallene D (**5**) in $CDCl_3$. S20
Figure S32. NOESY spectrum of thuwalallene D (**5**) in $CDCl_3$. S21
Figure S33. HR-APCIMS spectrum of thuwalallene D (**5**). S21
Figure S34. ^{1}H NMR spectrum of thuwalenyne B (**6**) in $CDCl_3$. S22
Figure S35. HSQC spectrum of thuwalenyne B (**6**) in $CDCl_3$. S22
Figure S36. HMBC spectrum of thuwalenyne B (**6**) in $CDCl_3$. S23
Figure S37. COSY spectrum of thuwalenyne B (**6**) in $CDCl_3$. S23
Figure S38. NOESY spectrum of thuwalenyne B (**6**) in $CDCl_3$. S24
Figure S39. HR-APCIMS spectrum of thuwalenyne B (**6**). S24
Figure S40. ^{1}H NMR spectrum of thuwalenyne C (**7**) in $CDCl_3$. S25
Figure S41. HSQC spectrum of thuwalenyne C (**7**) in $CDCl_3$. S25
Figure S42. HMBC spectrum of thuwalenyne C (**7**) in $CDCl_3$. S26
Figure S43. COSY spectrum of thuwalenyne C (**7**) in $CDCl_3$. S26
Figure S44. NOESY spectrum of thuwalenyne C (**7**) in $CDCl_3$. S27
Figure S45. HR-APCIMS spectrum of thuwalenyne C (**7**). S27
Figure S46. ^{1}H NMR spectrum of thuwalallene E (**8**) in $CDCl_3$. S28
Figure S47. ^{13}C NMR spectrum of thuwalallene E (**8**) in $CDCl_3$. S28
Figure S48. HSQC spectrum of thuwalallene E (**8**) in $CDCl_3$. S29
Figure S49. HMBC spectrum of thuwalallene E (**8**) in $CDCl_3$. S29
Figure S50. COSY spectrum of thuwalallene E (**8**) in $CDCl_3$. S30
Figure S51. NOESY spectrum of thuwalallene E (**8**) in $CDCl_3$. S30
Figure S52. HR-APCIMS spectrum of thuwalallene E (**8**). S31

Figure S53. ^{1}H NMR spectrum of *cis*-maneonene D (**9**) in $CDCl_3$. S32
Figure S54. ^{1}H NMR spectrum of thyrsiferol (**10**) in $CDCl_3$. S32
Figure S55. ^{1}H NMR spectrum of 23-acetyl-thyrsiferol (**11**) in $CDCl_3$. S33

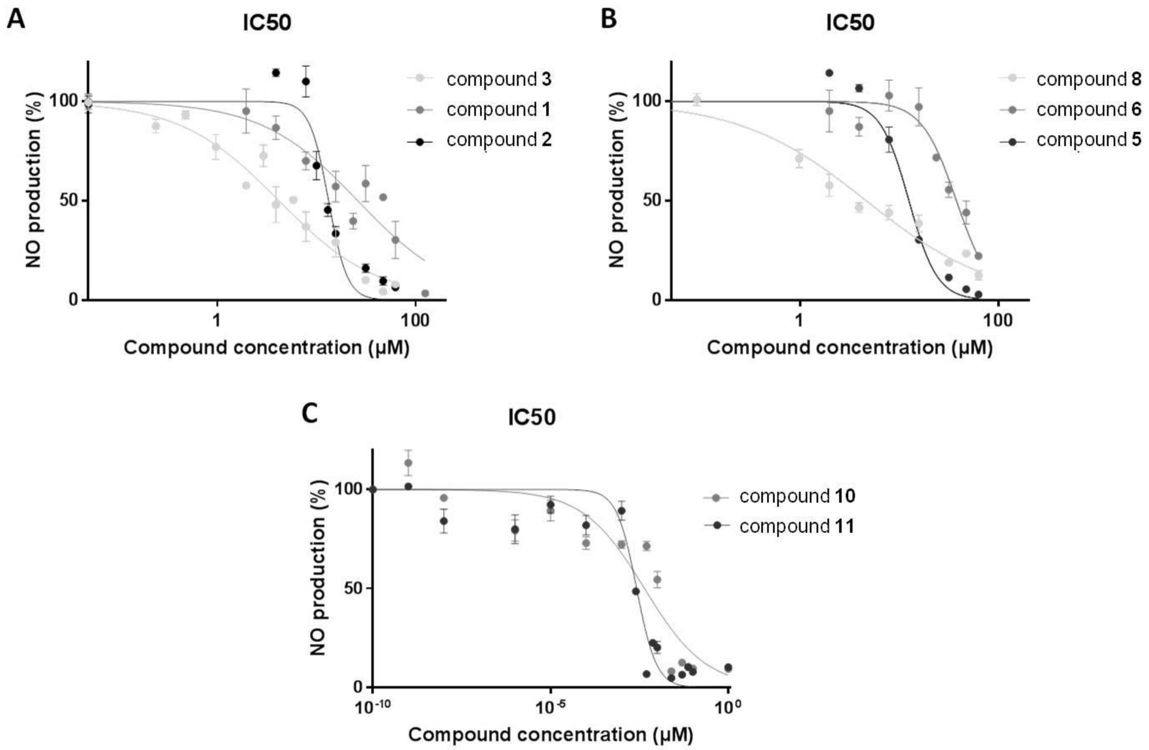

Figure S1. (**A**-**C**) Determination of concentration inducing 50% inhibition of NO production using LPS-treated RAW 264.7 for compounds **1–3**, **5**, **6**, **8**, **10** and **11**. Inhibition of NO production was compared to that of cells treated with Carbowax 400 0.1% v/v.

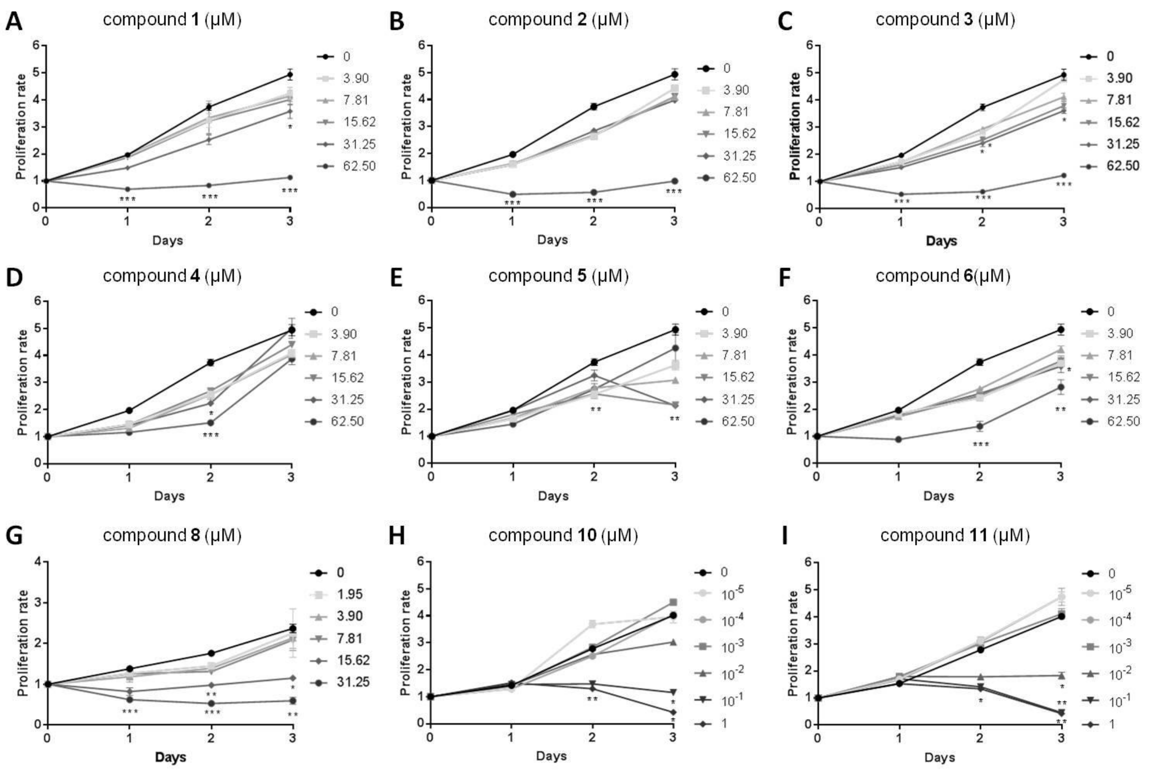

Figure S2. (**A**-**I**) Evaluation of the cytotoxic activity of compounds **1–6**, **8**, **10** and **11** by measuring proliferation rate of RAW 264.7 cells. Proliferation rate was established using MTT treatment for 72 h and normalized to measurement of the initial cells plated and compared to that of cells treated with Carbowax 400 0.1% v/v. Statistical analysis was performed using Kruskal-Walis non-parametric test in Graphpad Prism 7.0. Graphs represent mean ± SEM (* indicates $P<0.05$, **indicates $P<0.01$, ***indicates $P<0.001$).

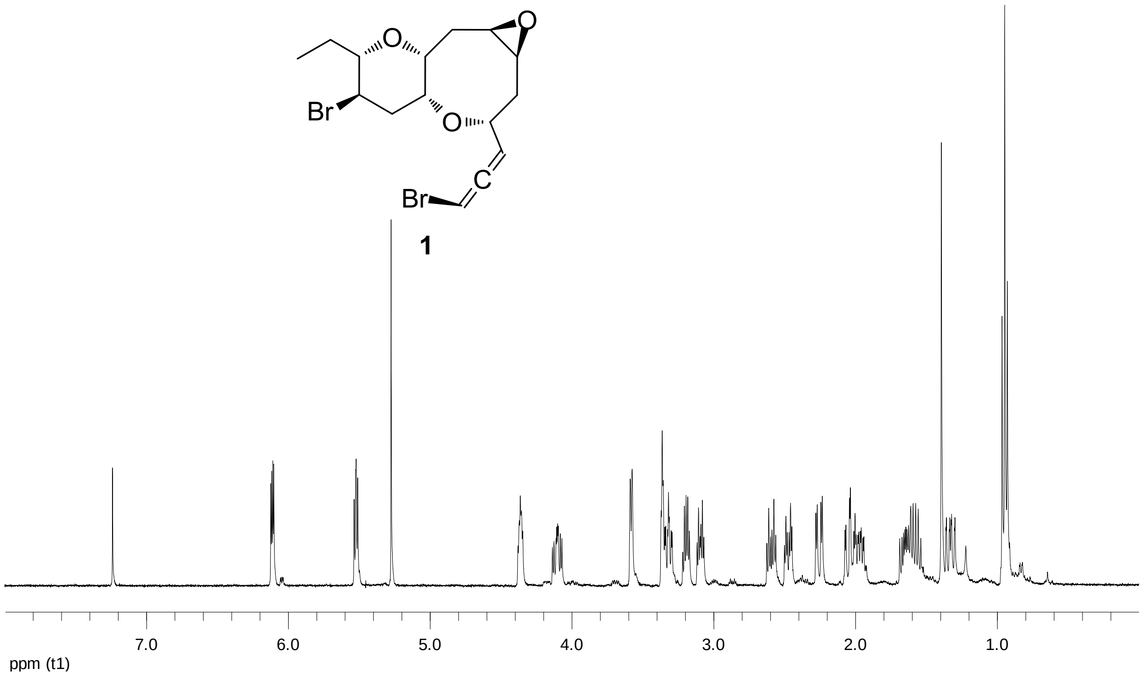

Figure S3. ^{1}H NMR spectrum of thuwalallene A (**1**) in $CDCl_3$.

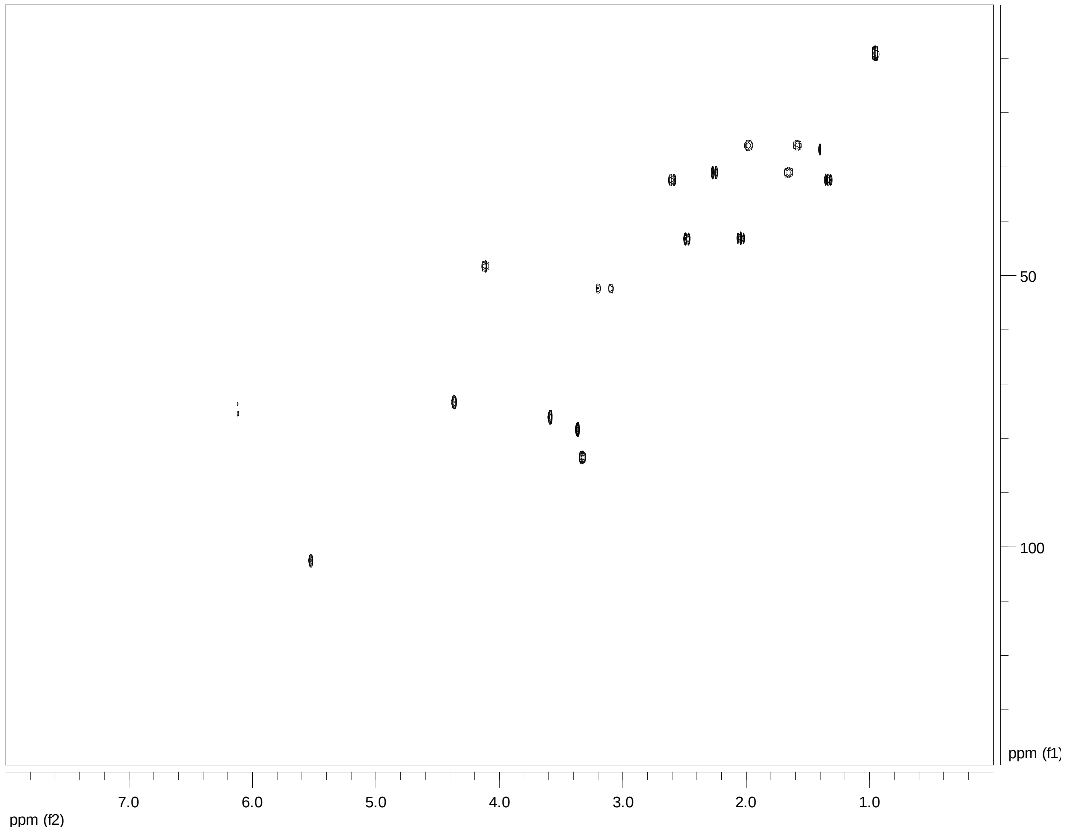

Figure S4. HSQC spectrum of thuwalallene A (**1**) in $CDCl_3$.

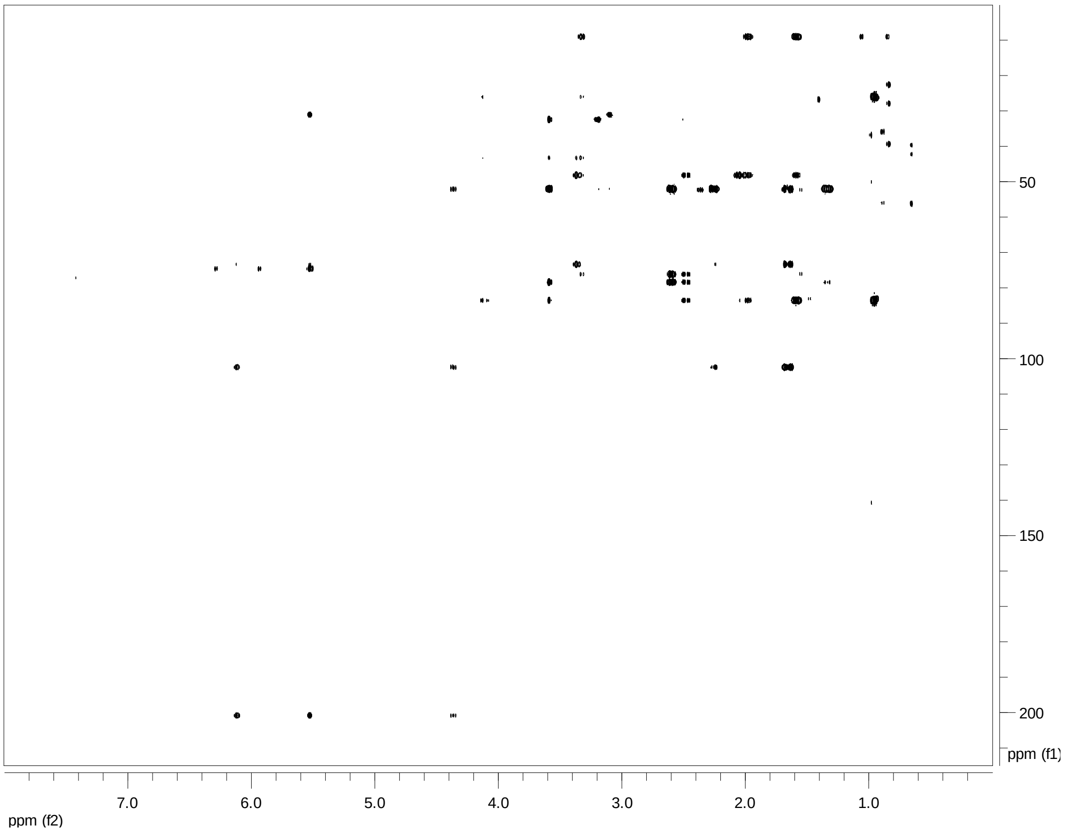

Figure S5. HMBC spectrum of thuwalallene A (**1**) in $CDCl_3$.

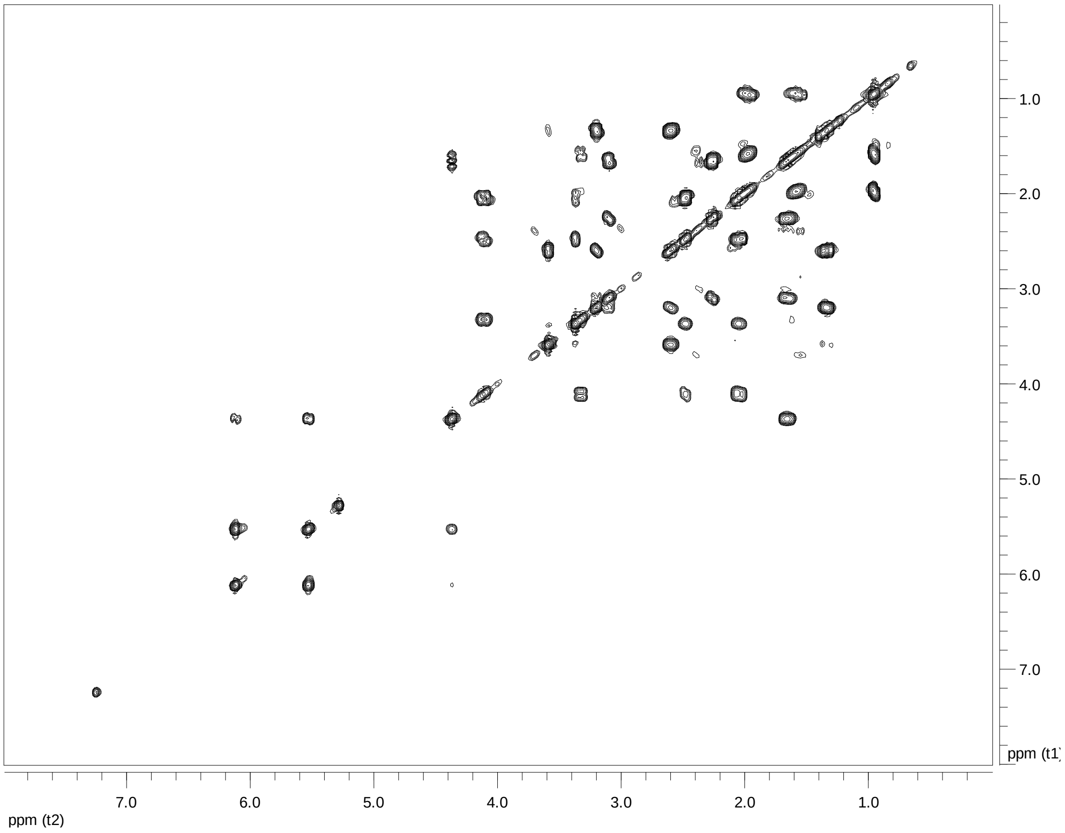

Figure S6. COSY spectrum of thuwalallene A (**1**) in $CDCl_3$.

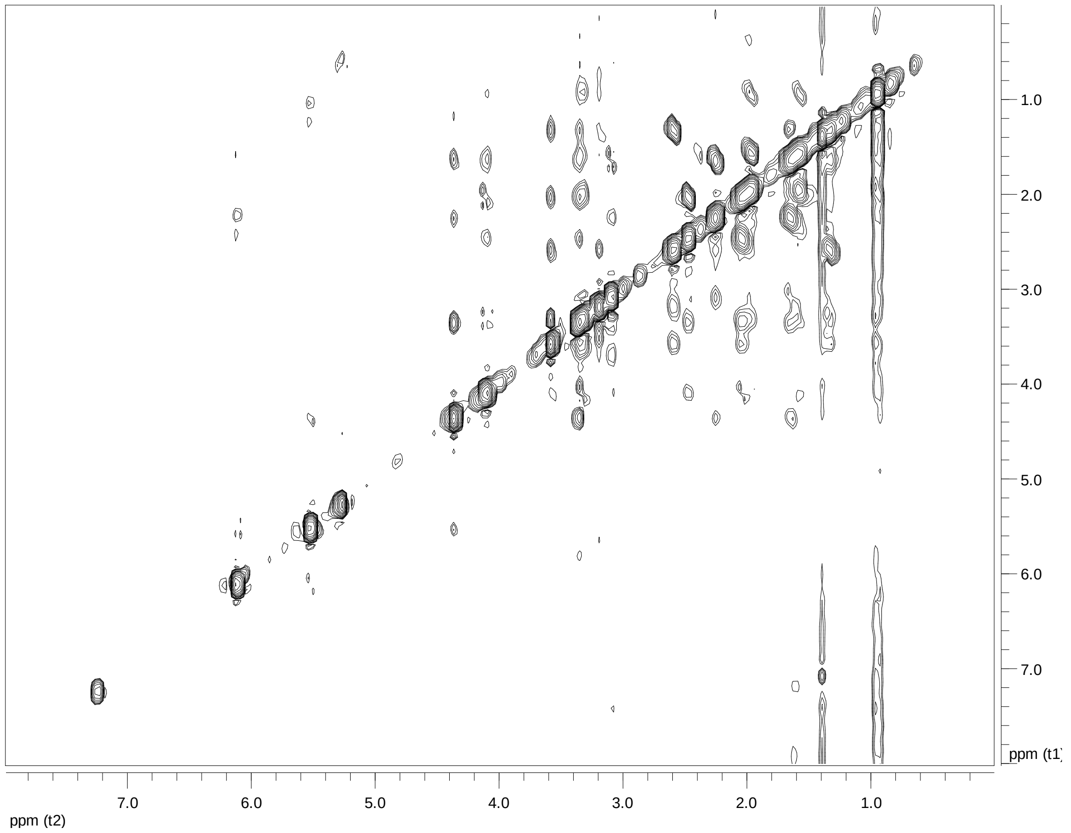

Figure S7. NOESY spectrum of thuwalallene A (**1**) in $CDCl_3$.

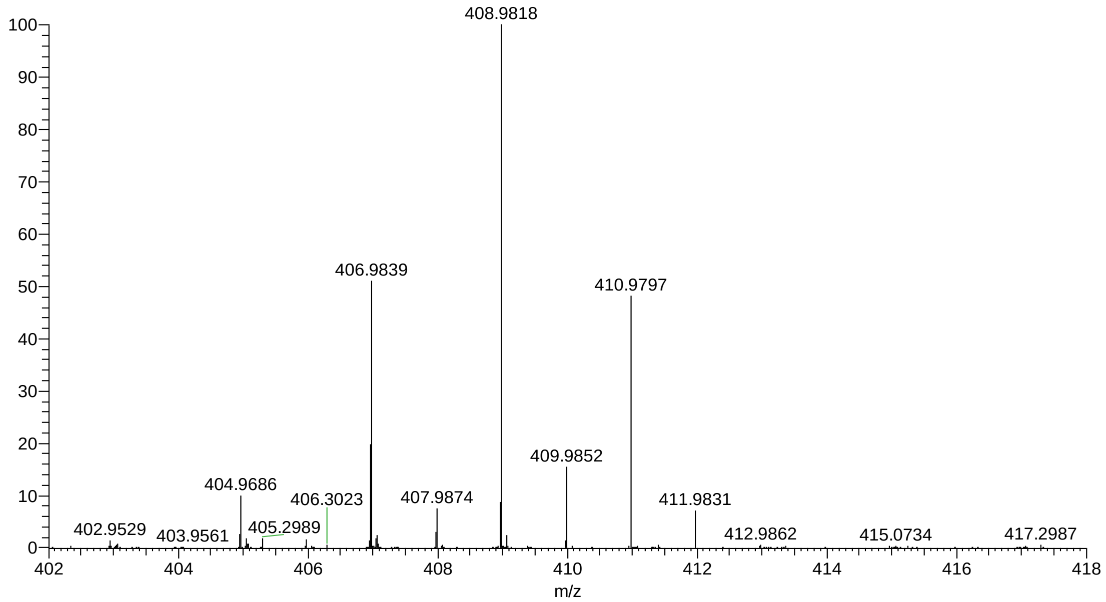

Figure S8. HR-APCIMS spectrum of thuwalallene A (**1**).

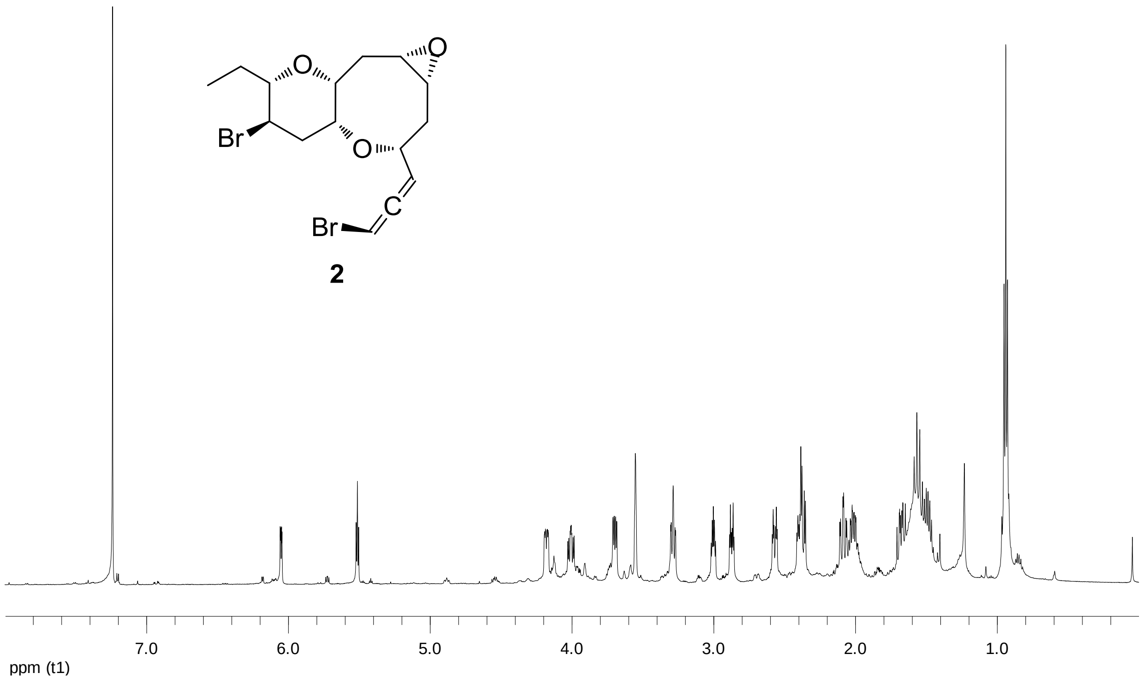

Figure S9. ^{1}H NMR spectrum of thuwalallene B (**2**) in $CDCl_3$.

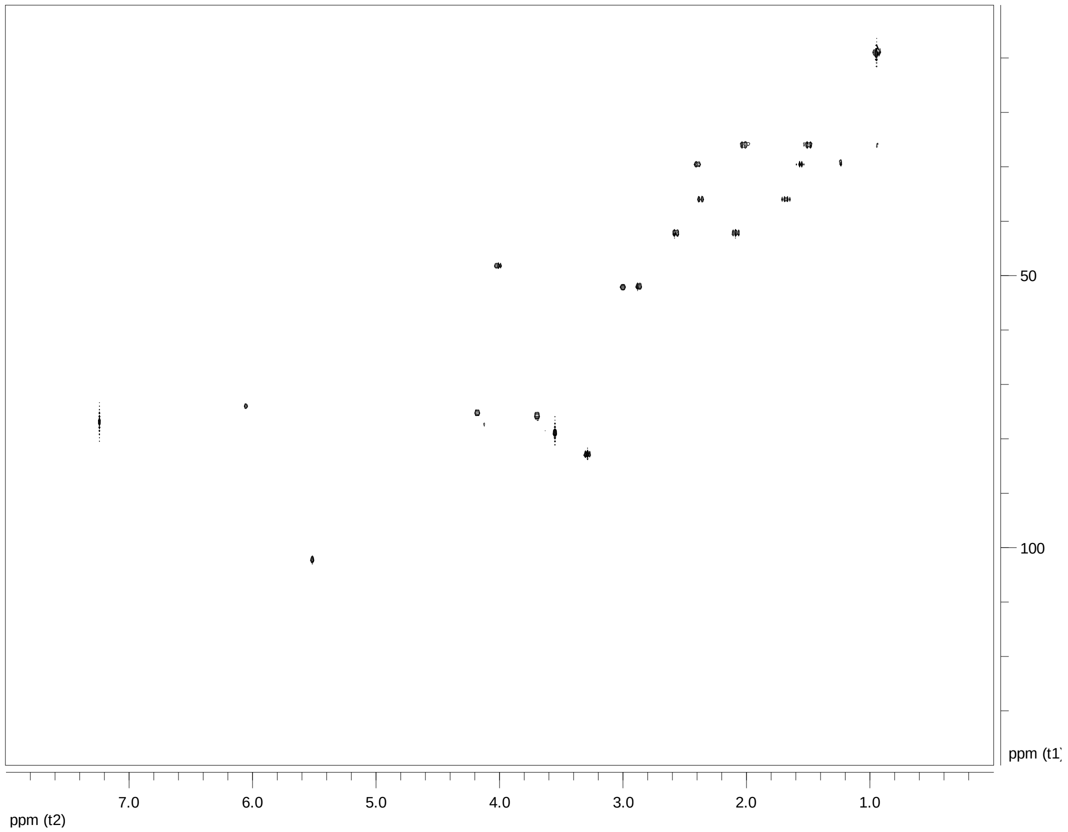

Figure S10. HSQC spectrum of thuwalallene B (**2**) in $CDCl_3$.

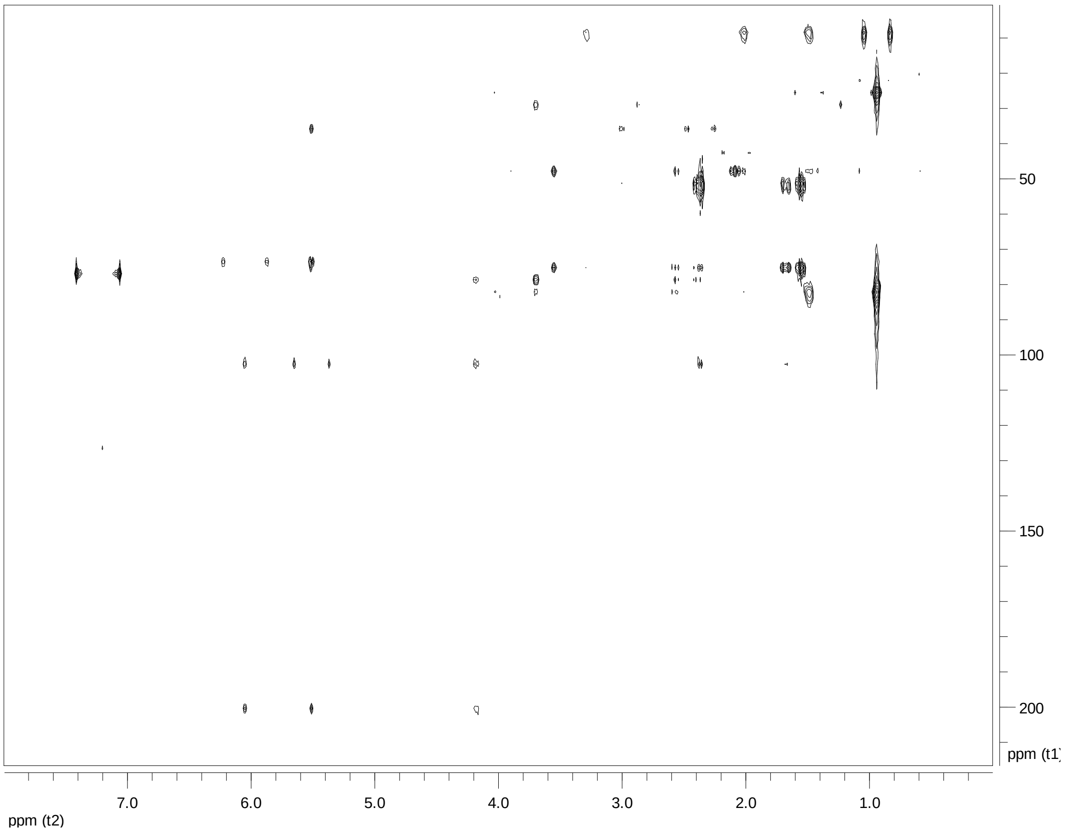

Figure S11. HMBC spectrum of thuwalallene B (**2**) in $CDCl_3$.

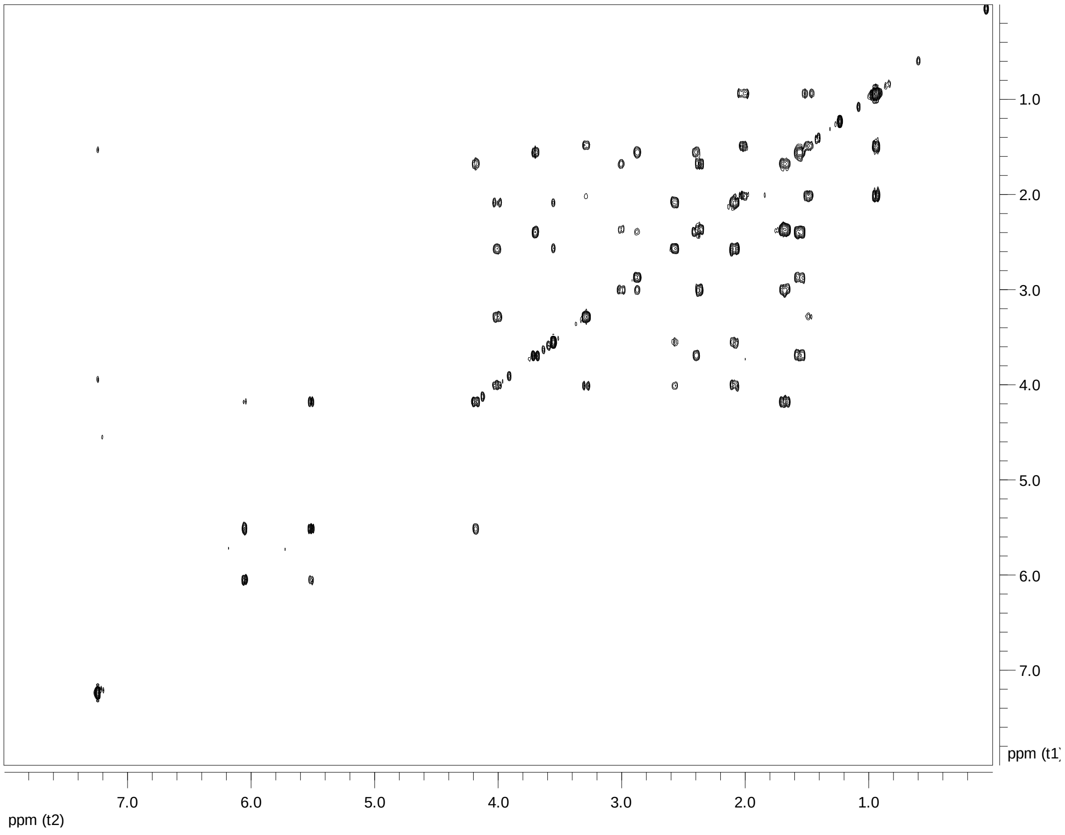

Figure S12. COSY spectrum of thuwalallene B (**2**) in $CDCl_3$.

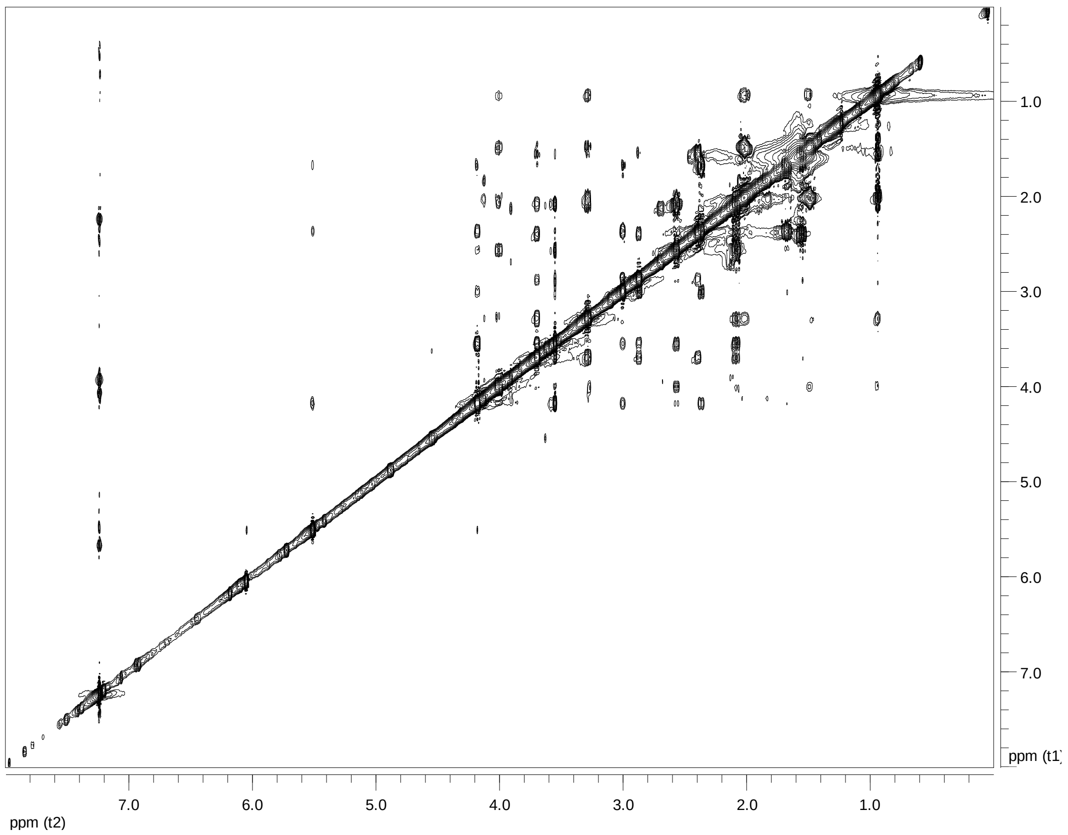

Figure S13. NOESY spectrum of thuwalallene B (**2**) in $CDCl_3$.

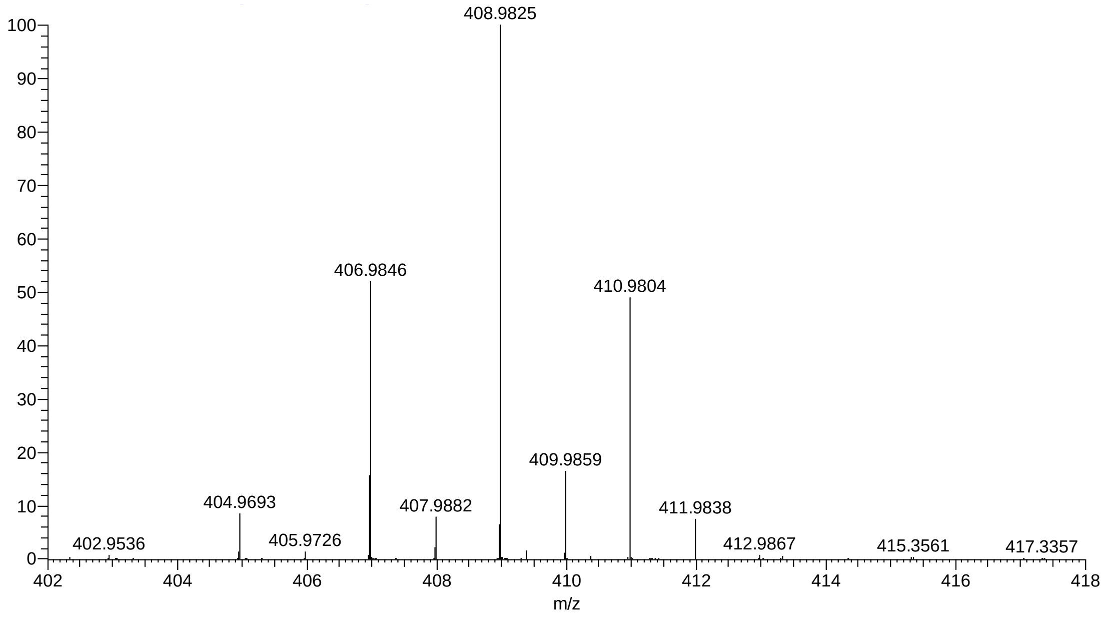

Figure S14. HR-APCIMS spectrum of thuwalallene B (**2**).

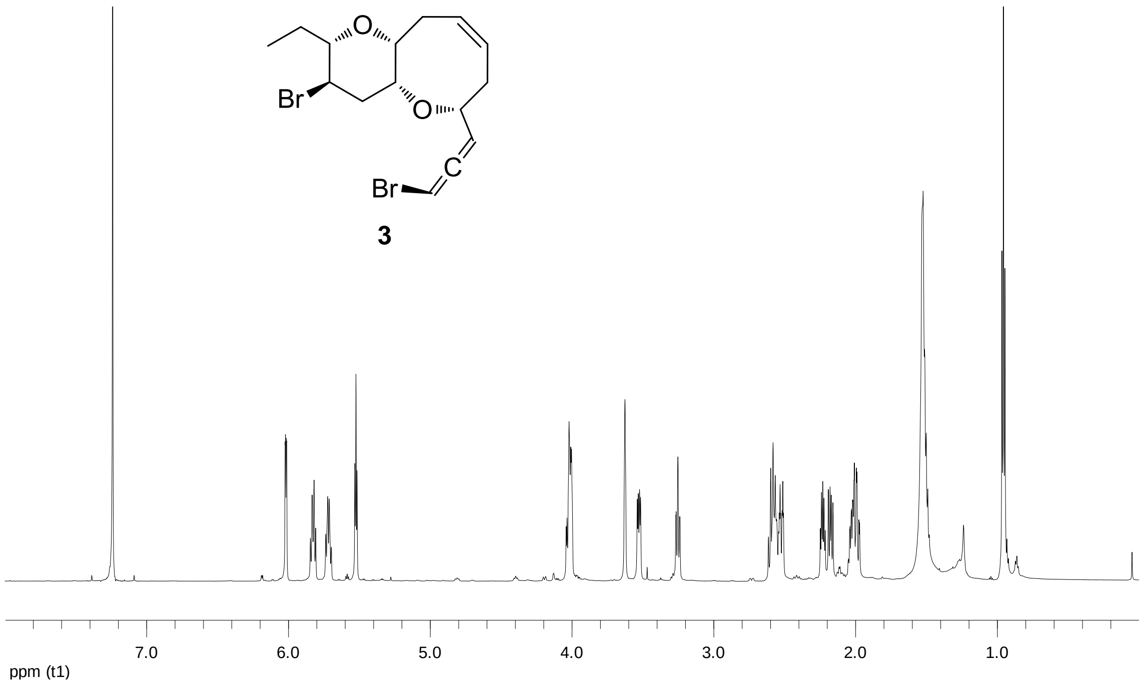

Figure S15. ^{1}H NMR spectrum of thuwalallene C (**3**) in $CDCl_3$.

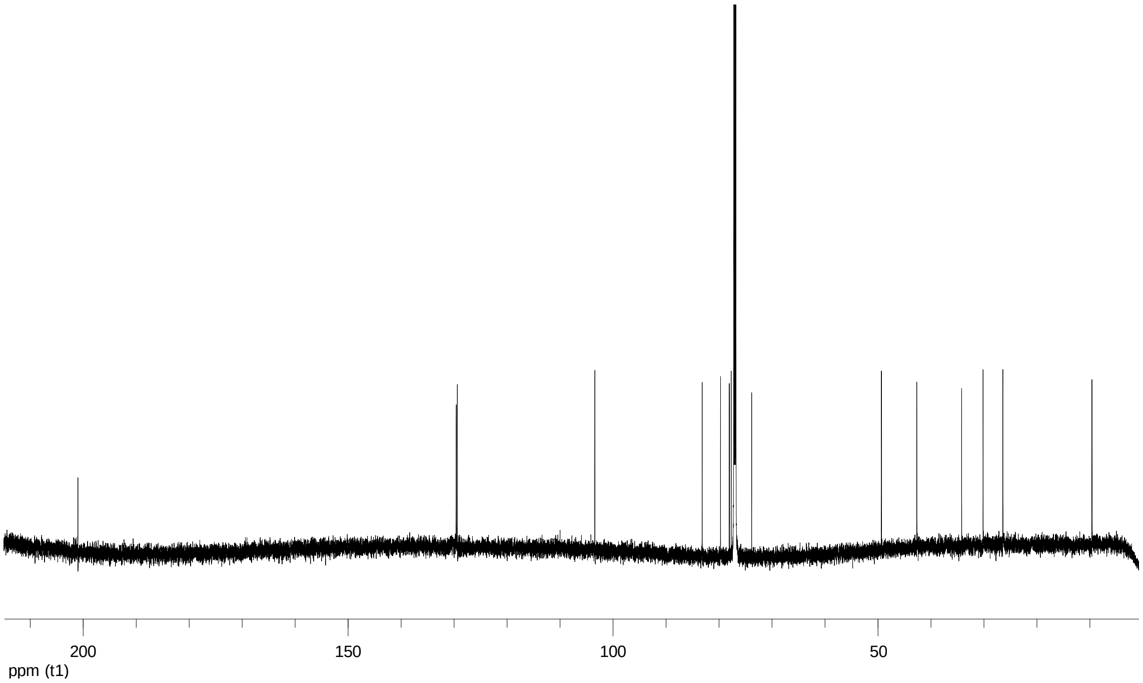

Figure S16. ^{13}C NMR spectrum of thuwalallene C (**3**) in $CDCl_3$.

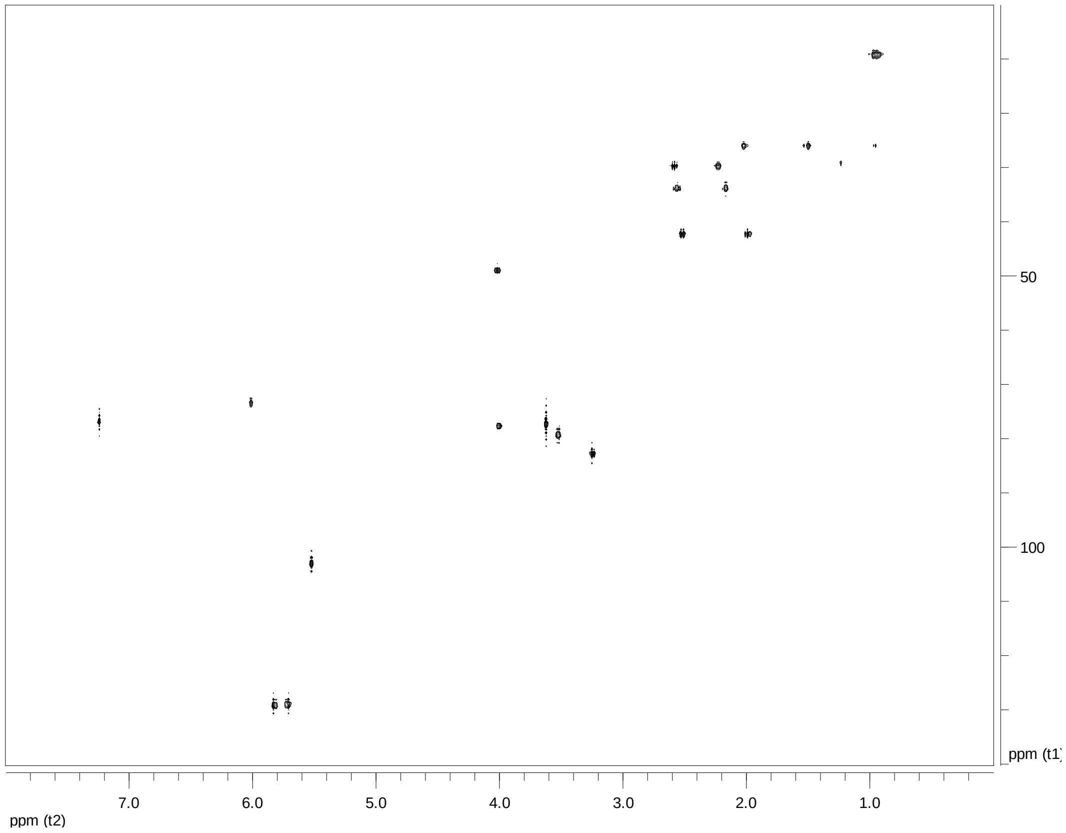

Figure S17. HSQC spectrum of thuwalallene C (**3**) in $CDCl_3$.

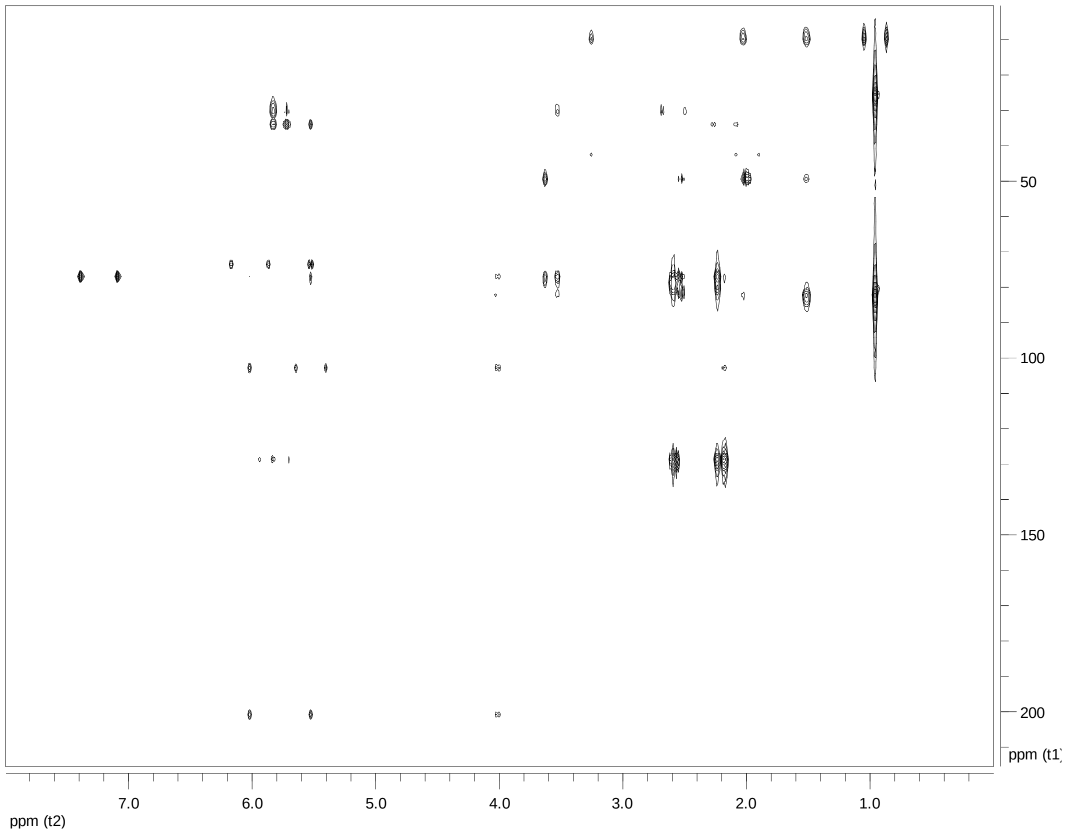

Figure S18. HMBC spectrum of thuwalallene C (**3**) in $CDCl_3$.

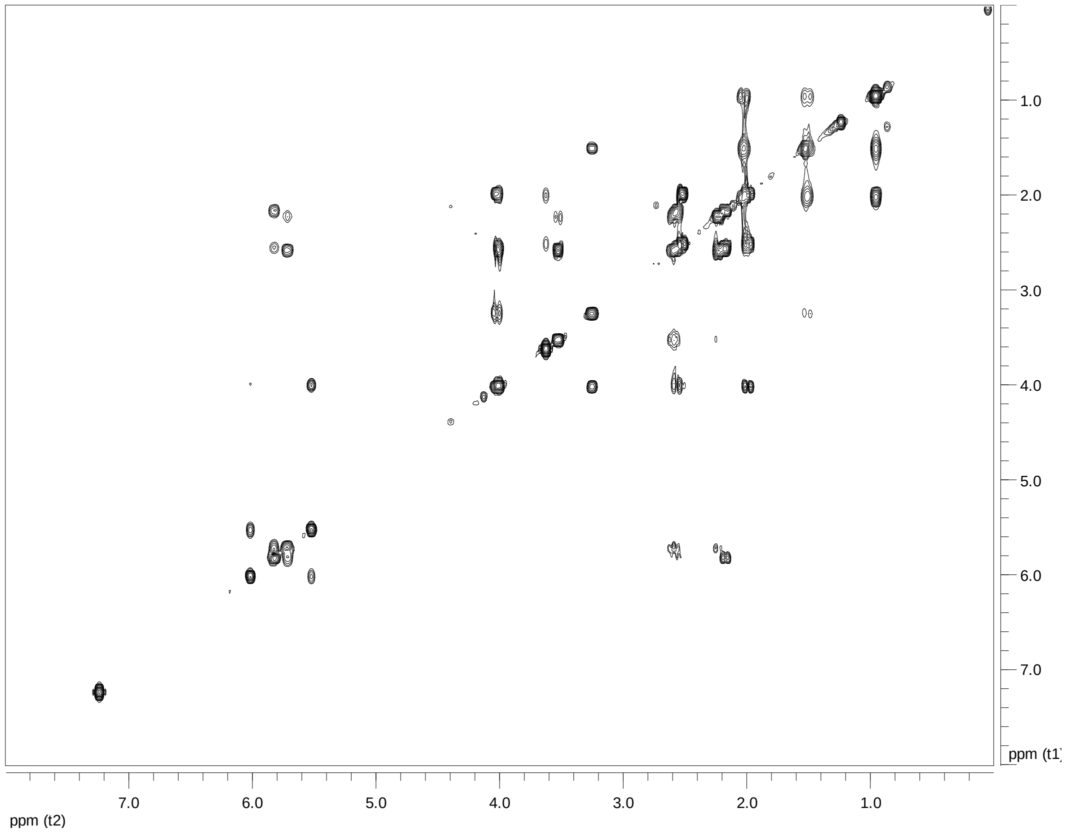

Figure S19. COSY spectrum of thuwalallene C (**3**) in $CDCl_3$.

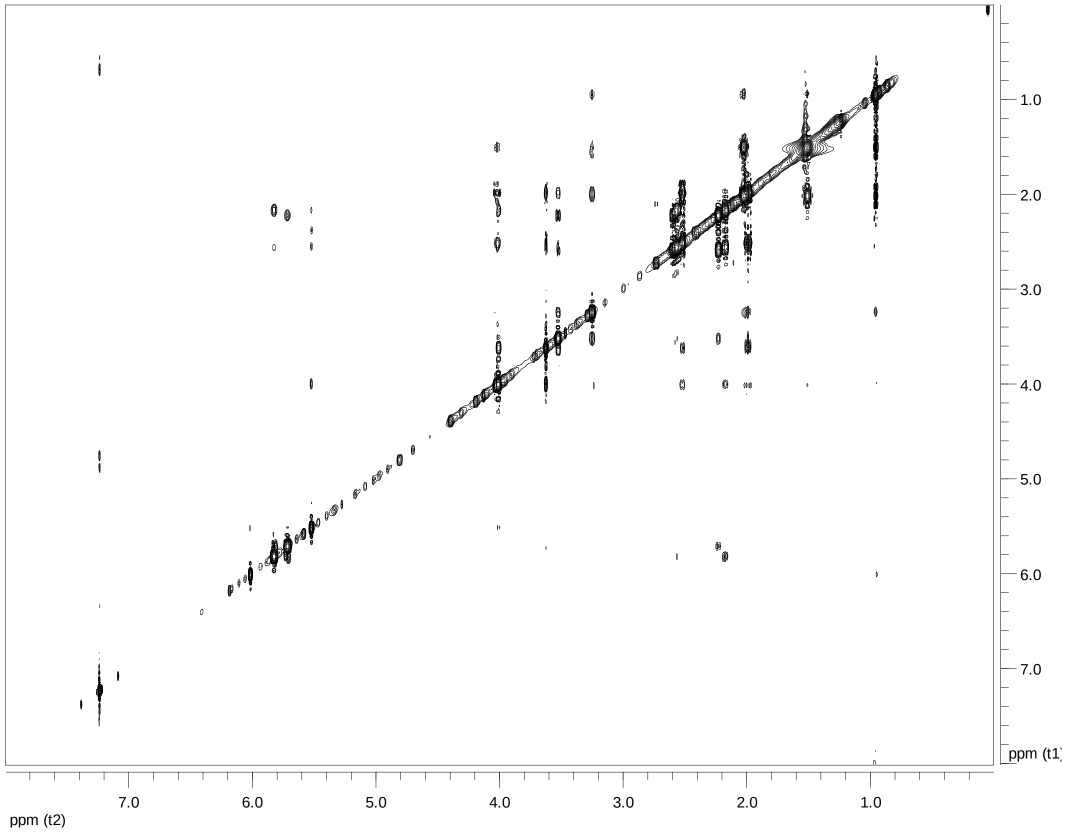

Figure S20. NOESY spectrum of thuwalallene C (**3**) in $CDCl_3$.

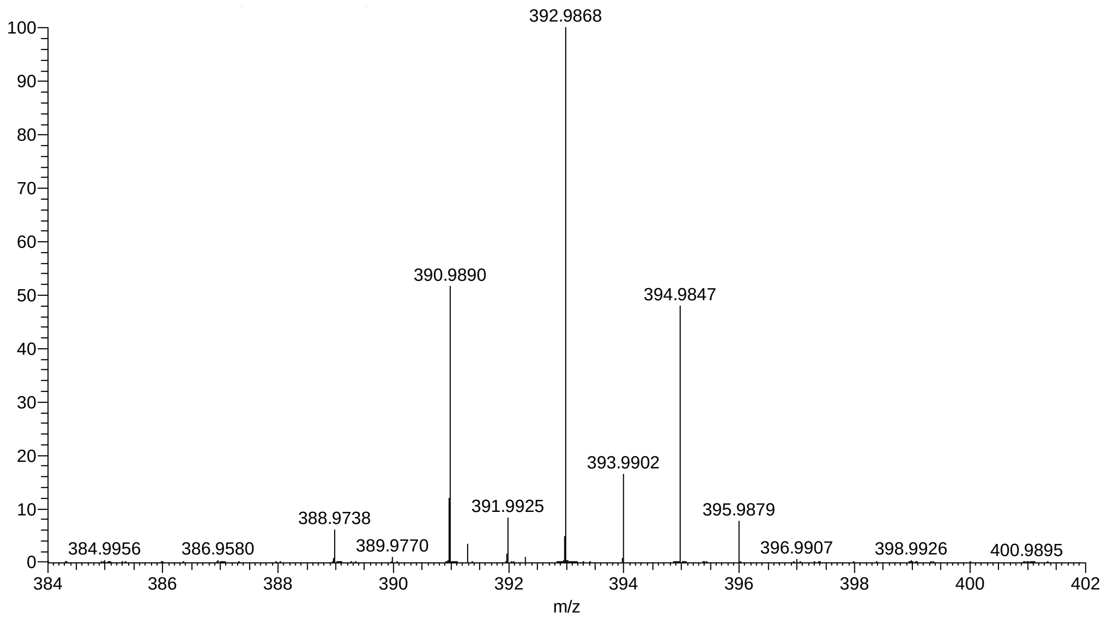

Figure S21. HR-APCIMS spectrum of thuwalallene C (**3**).

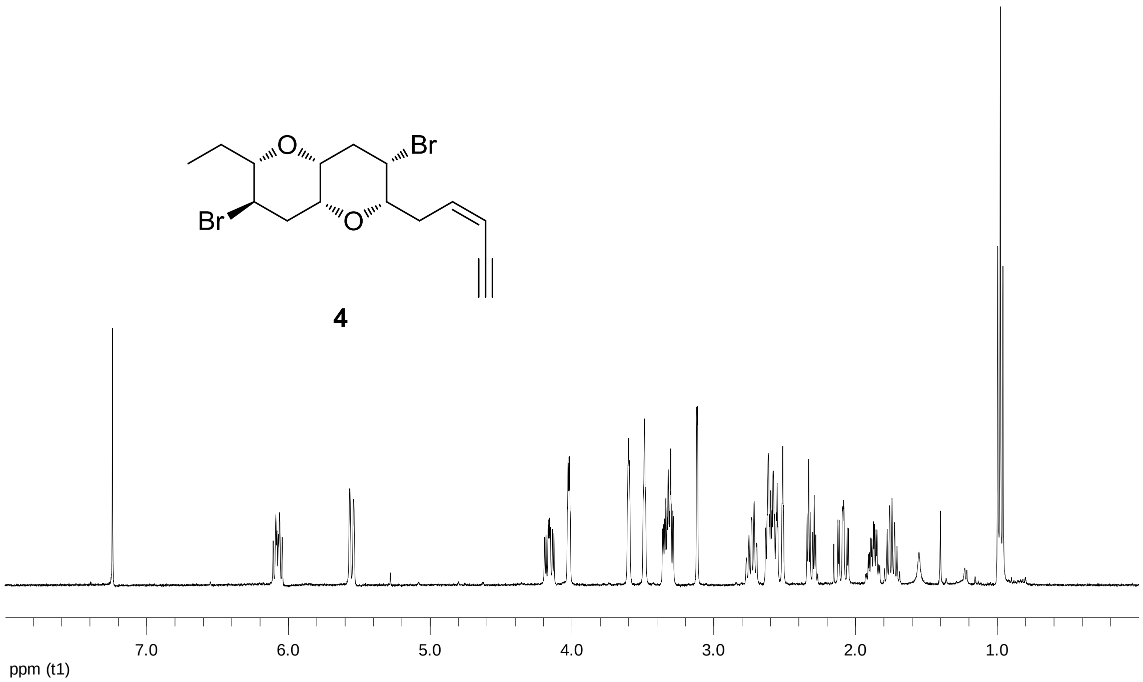

Figure S22. ^{1}H NMR spectrum of thuwalenyne A (**4**) in $CDCl_3$.

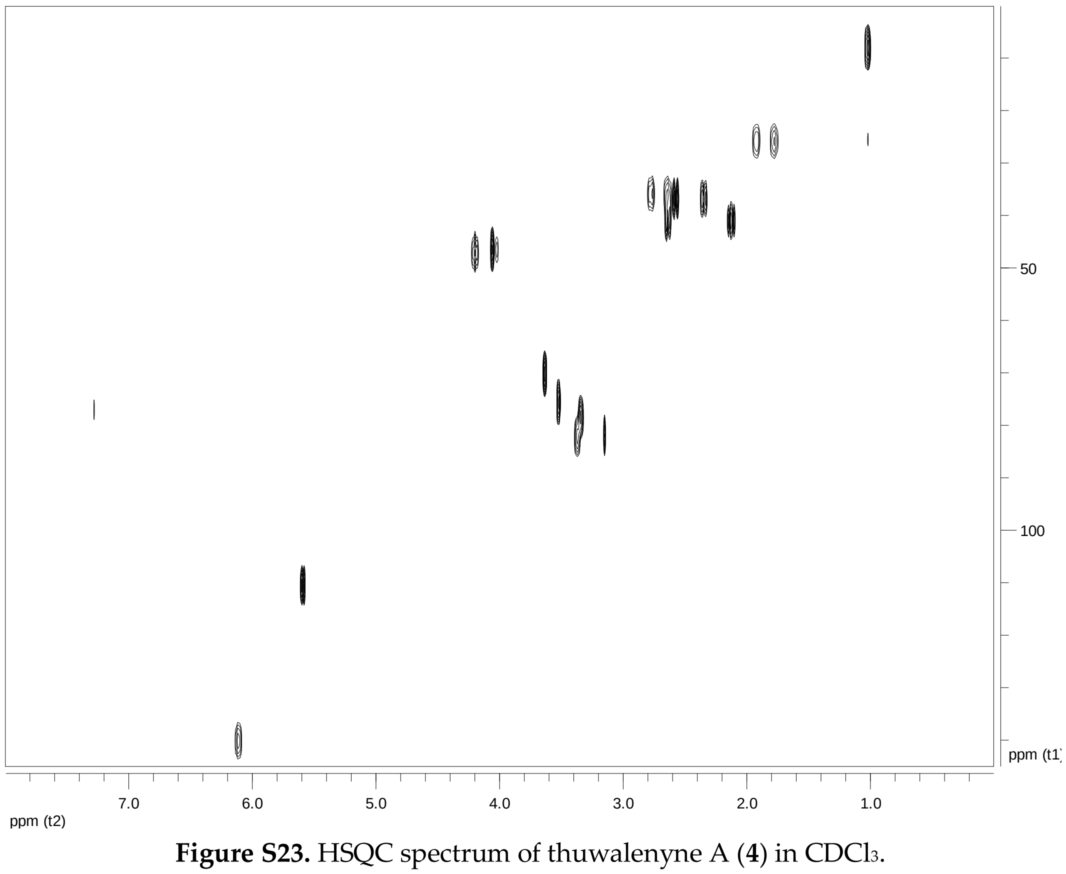

Figure S23. HSQC spectrum of thuwalenyne A (**4**) in $CDCl_3$.

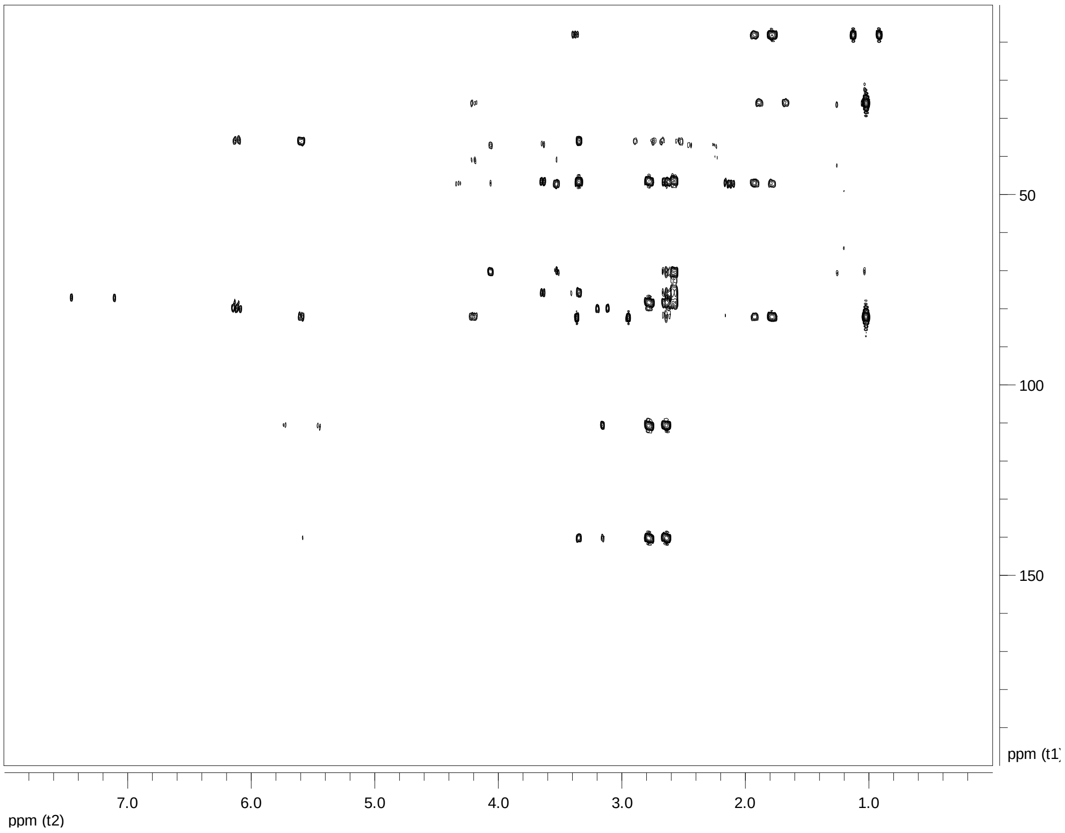

Figure S24. HMBC spectrum of thuwalenyne A (**4**) in $CDCl_3$.

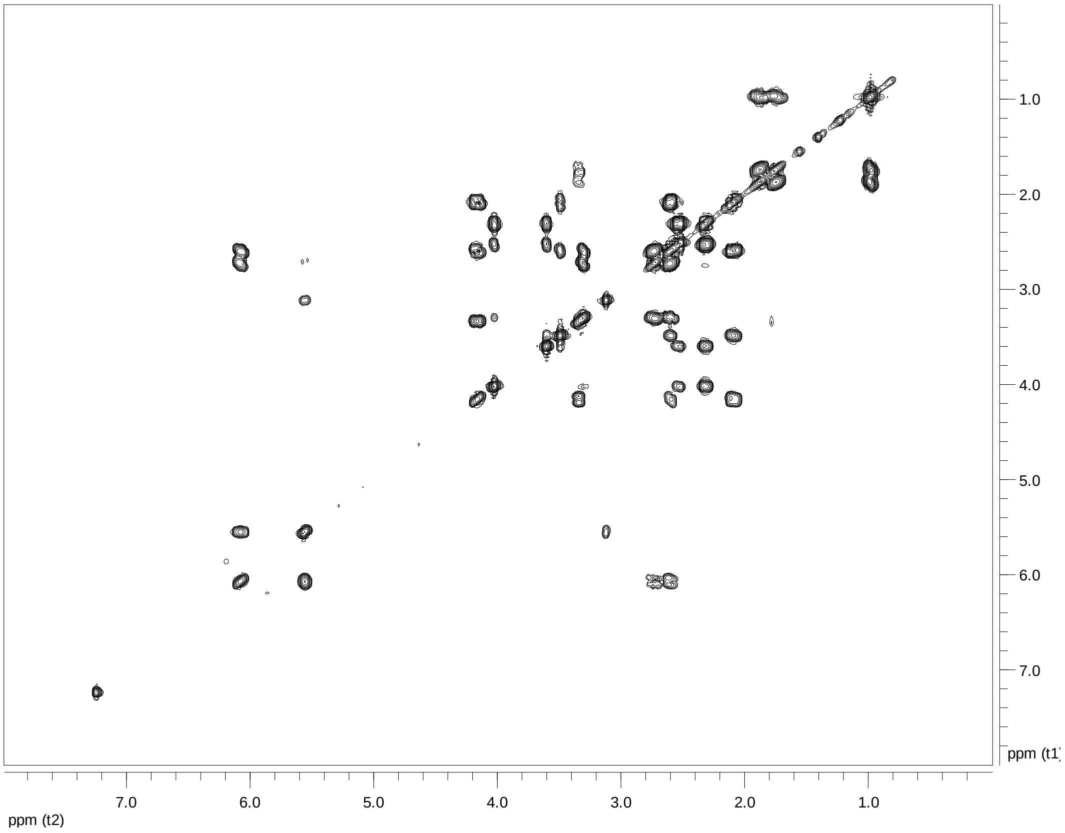

Figure S25. COSY spectrum of thuwalenyne A (**4**) in $CDCl_3$.

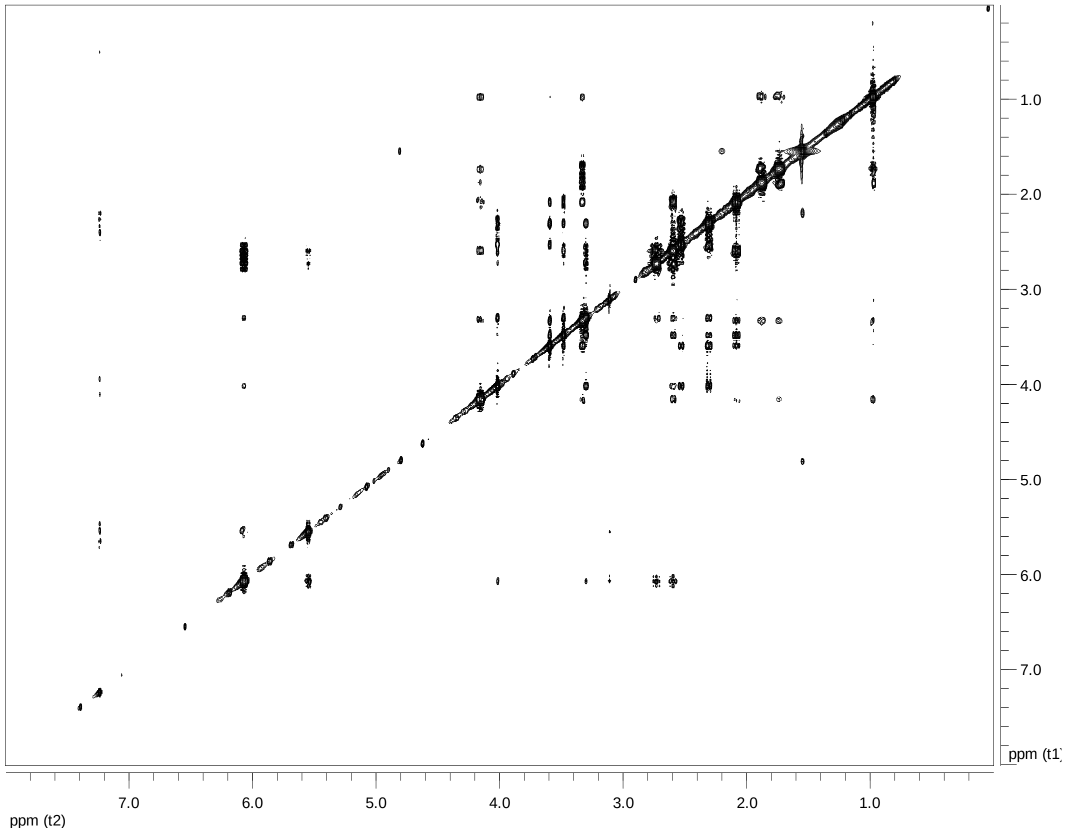

Figure S26. NOESY spectrum of thuwalenyne A (**4**) in $CDCl_3$.

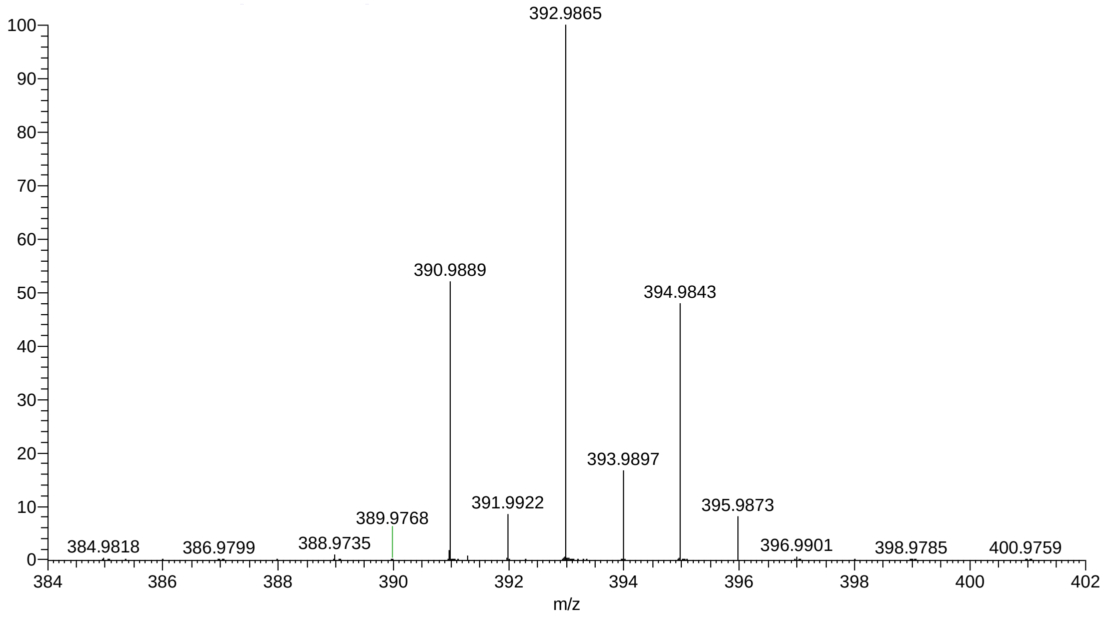

Figure S27. HR-APCIMS spectrum of thuwalenyne A (**4**).

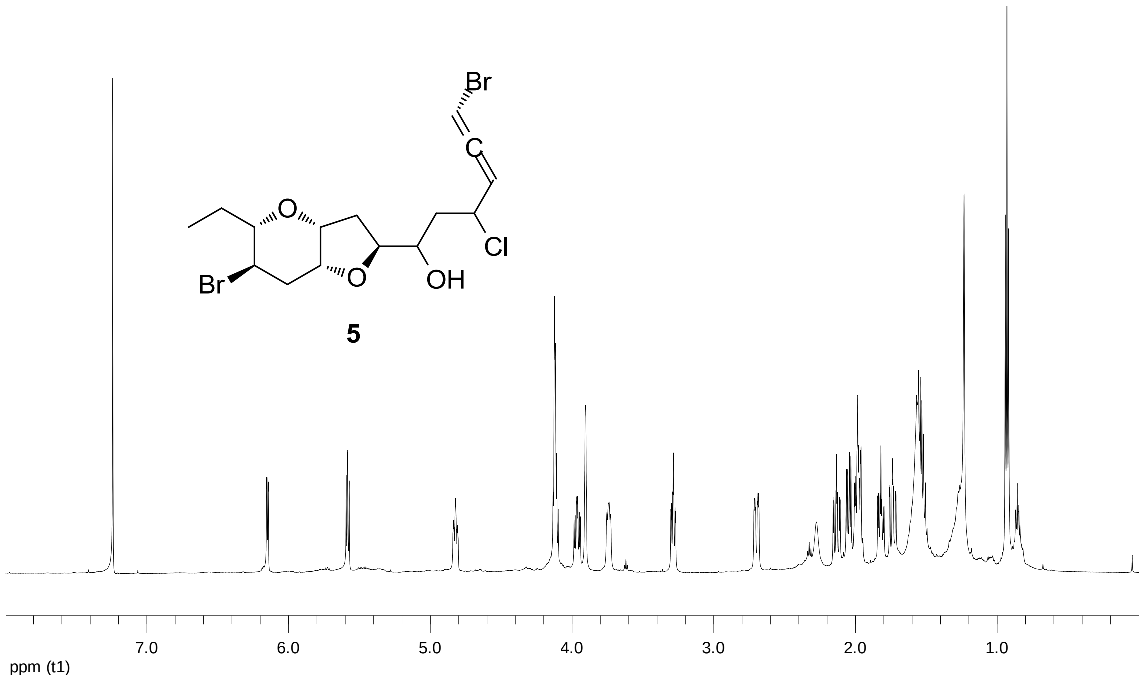

Figure S28. ^{1}H NMR spectrum of thuwalallene D (**5**) in $CDCl_3$.

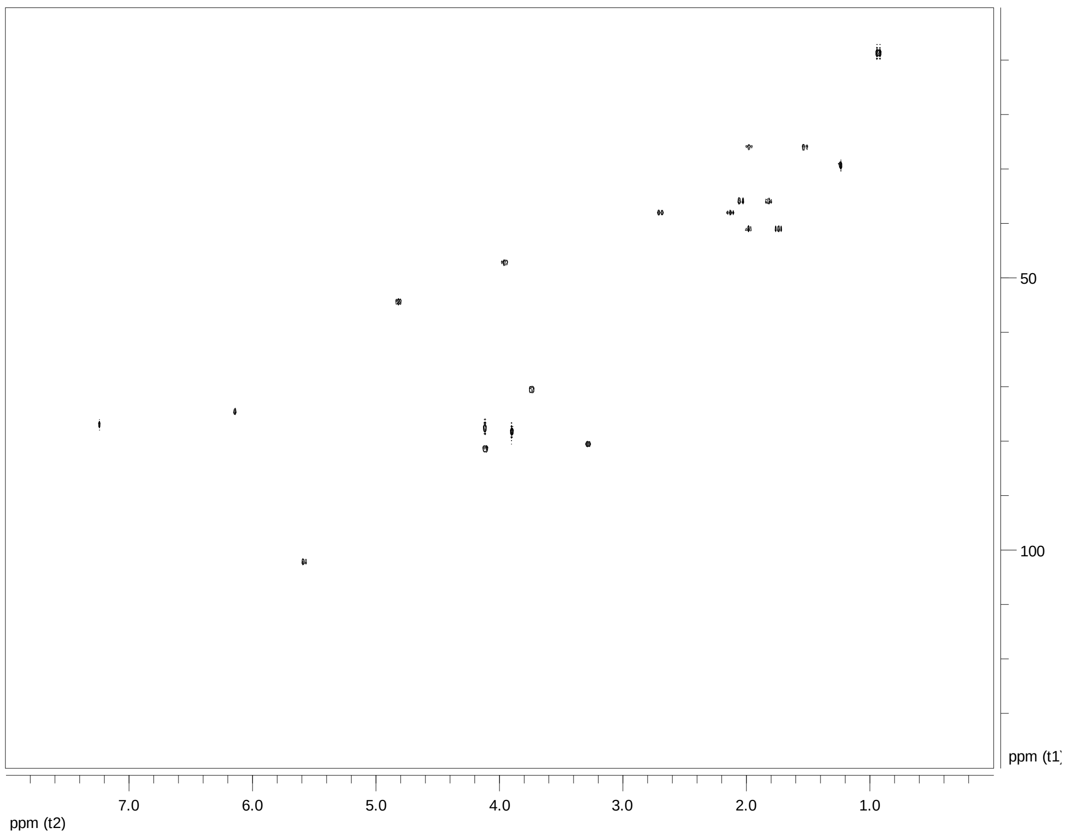

Figure S29. HSQC spectrum of thuwalallene D (**5**) in $CDCl_3$.

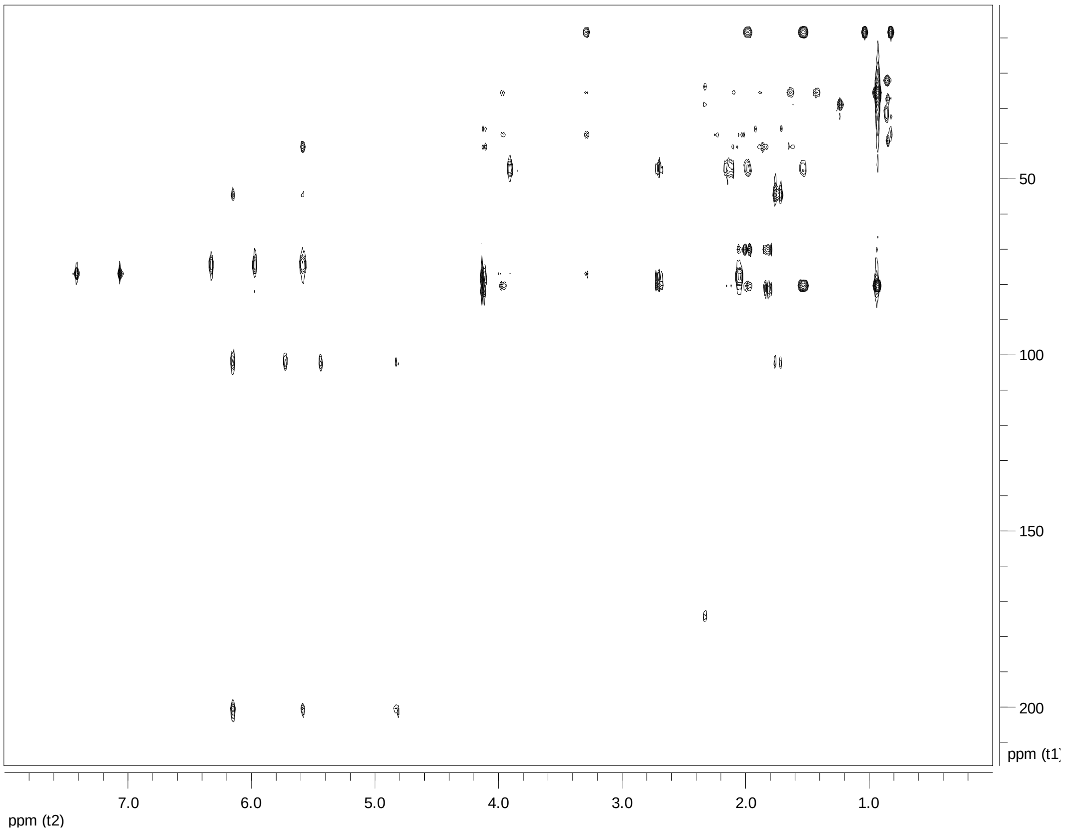

Figure S30. HMBC spectrum of thuwalallene D (**5**) in $CDCl_3$.

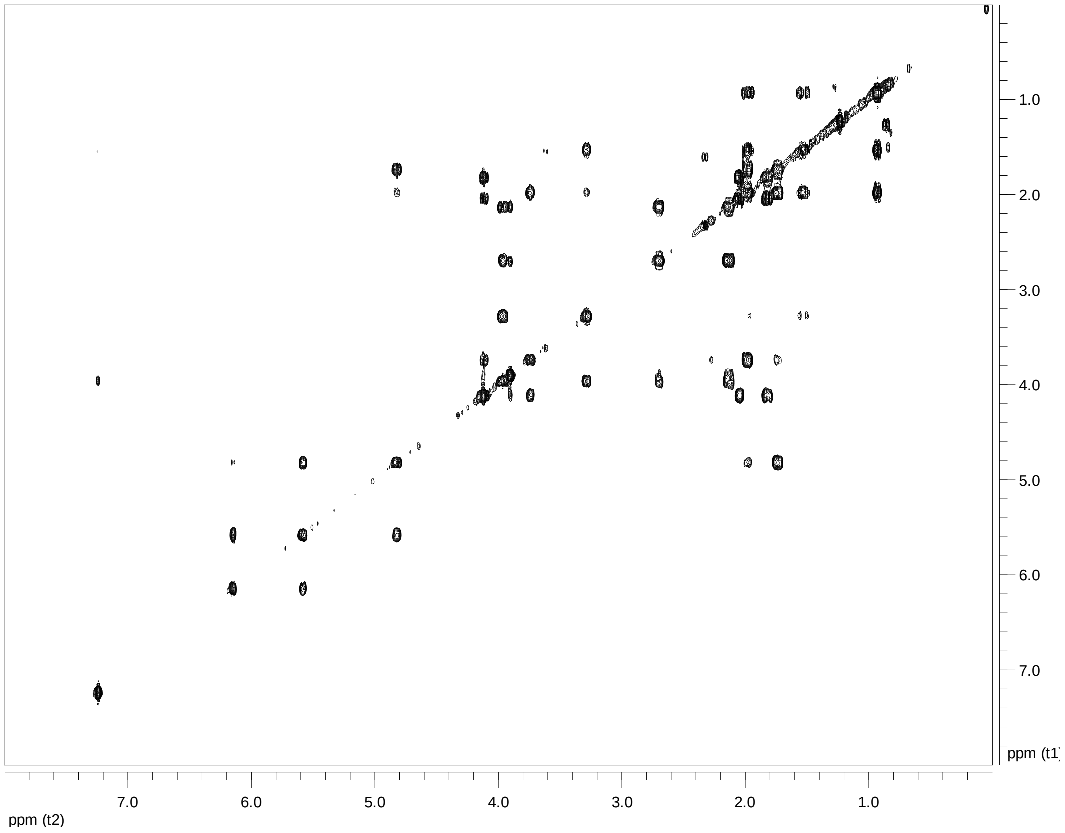

Figure S31. COSY spectrum of thuwalallene D (**5**) in $CDCl_3$.

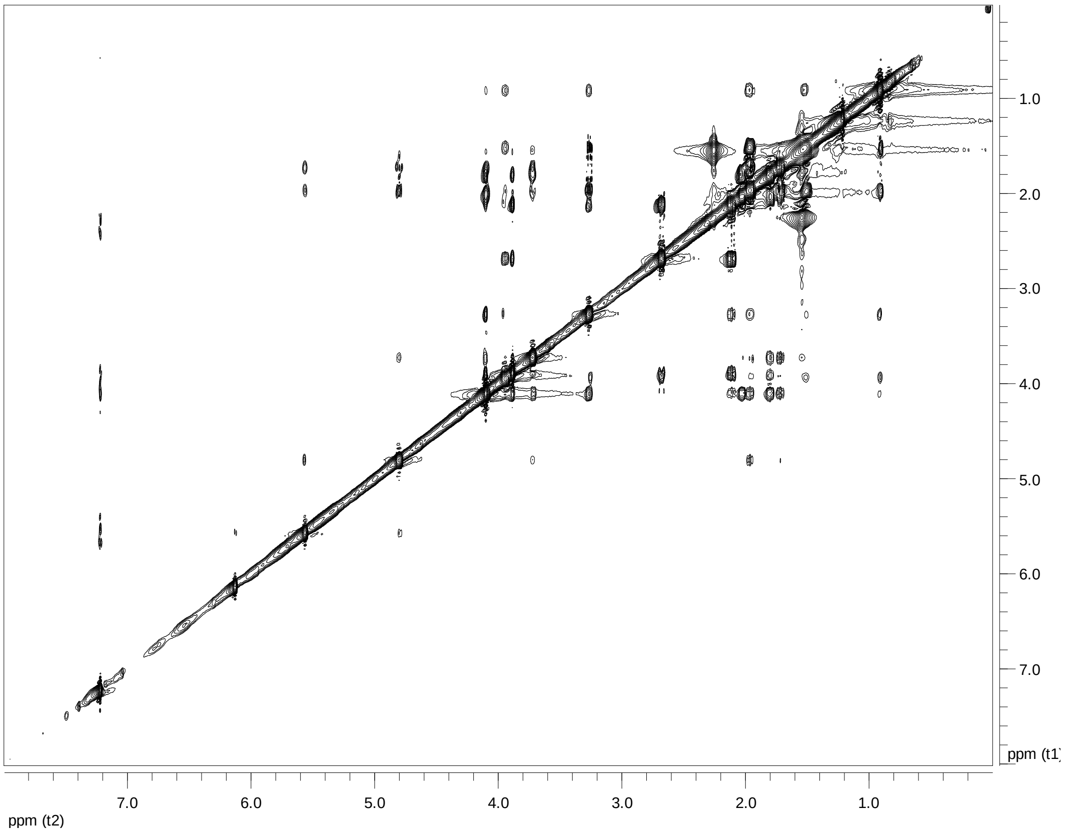

Figure S32. NOESY spectrum of thuwalallene D (**5**) in $CDCl_3$.

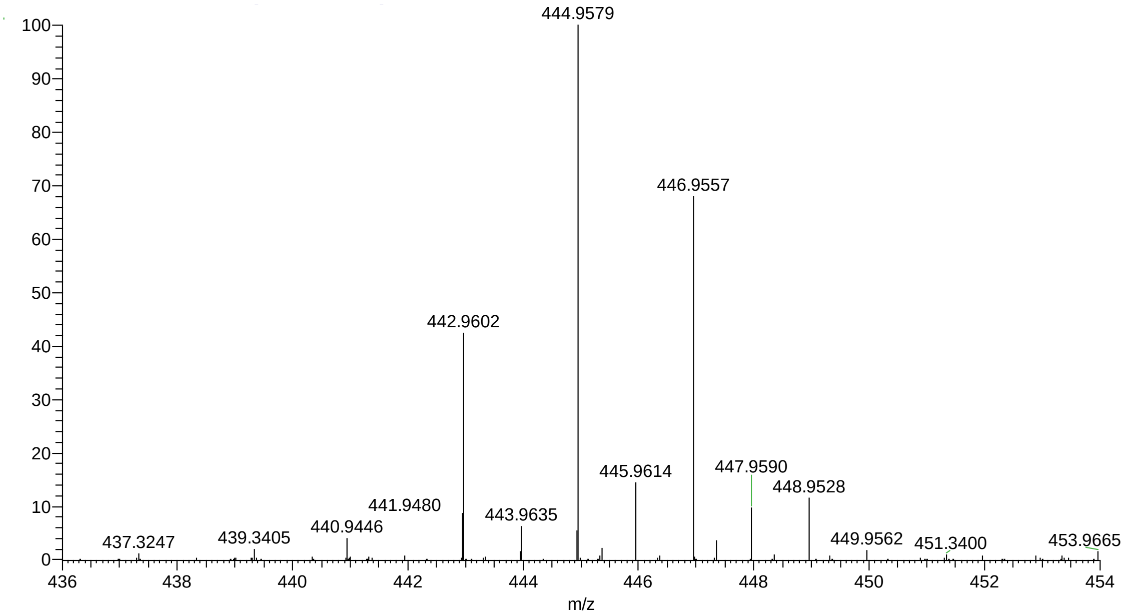

Figure S33. HR-APCIMS spectrum of thuwalallene D (**5**).

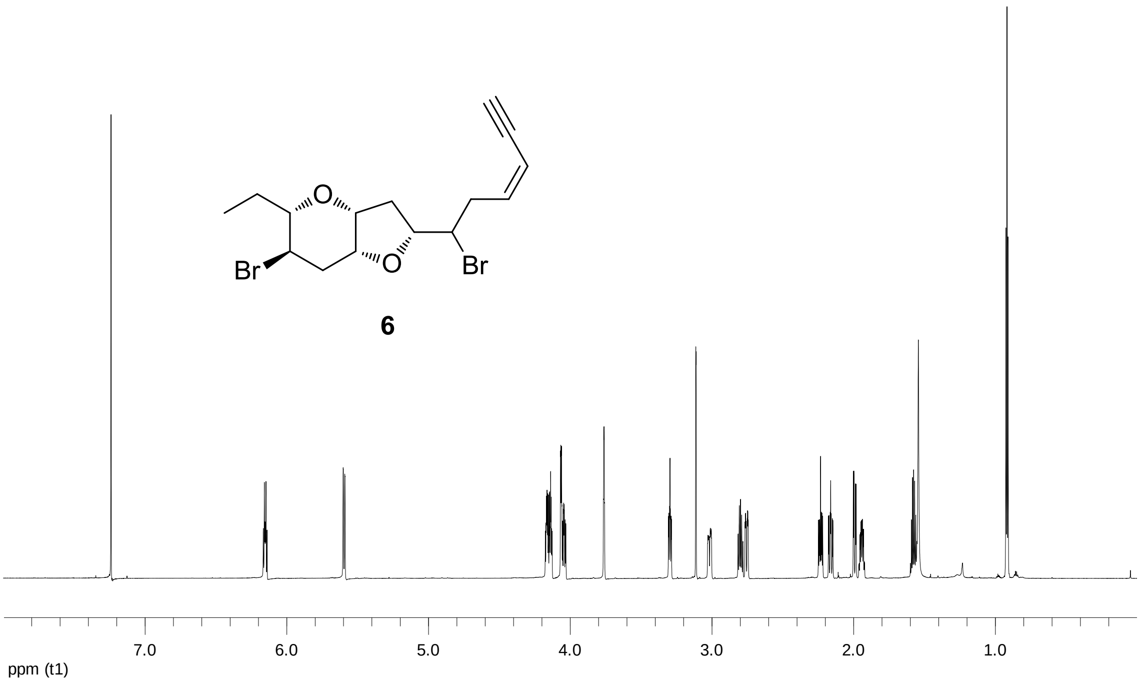

Figure S34. ^{1}H NMR spectrum of thuwalenyne B (**6**) in $CDCl_3$.

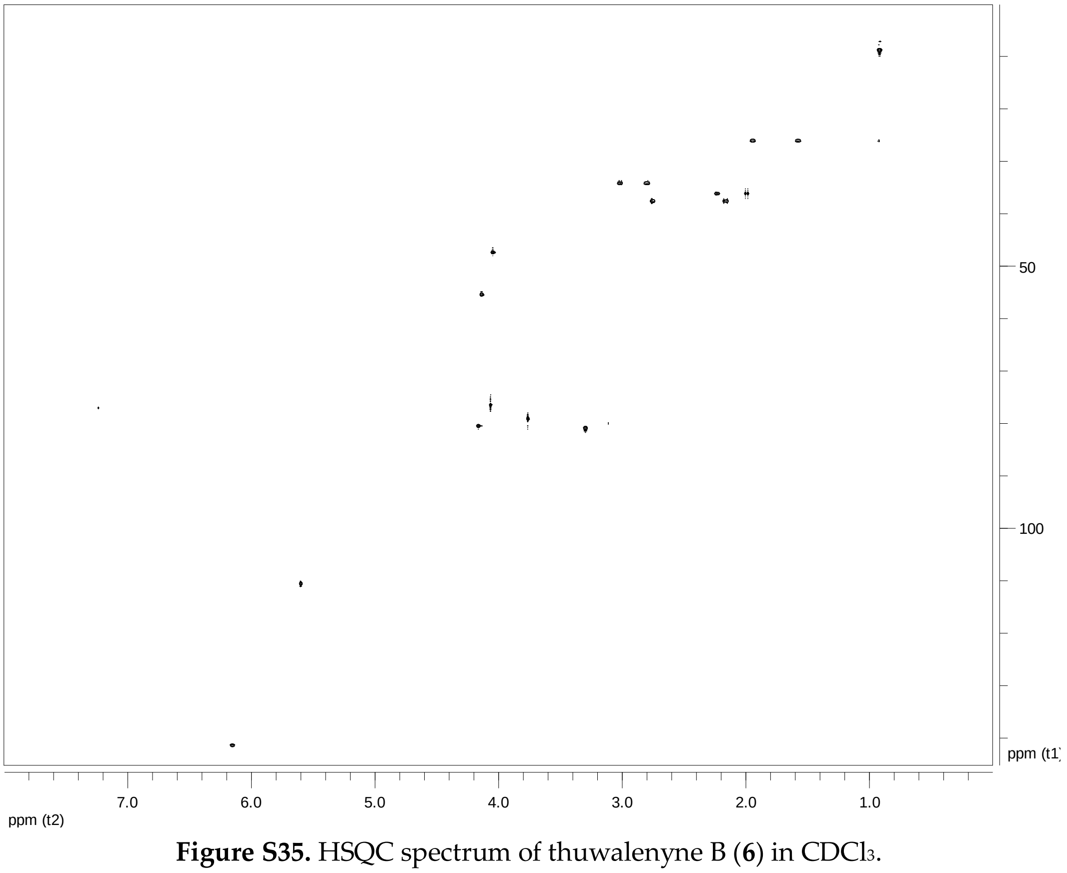

Figure S35. HSQC spectrum of thuwalenyne B (**6**) in $CDCl_3$.

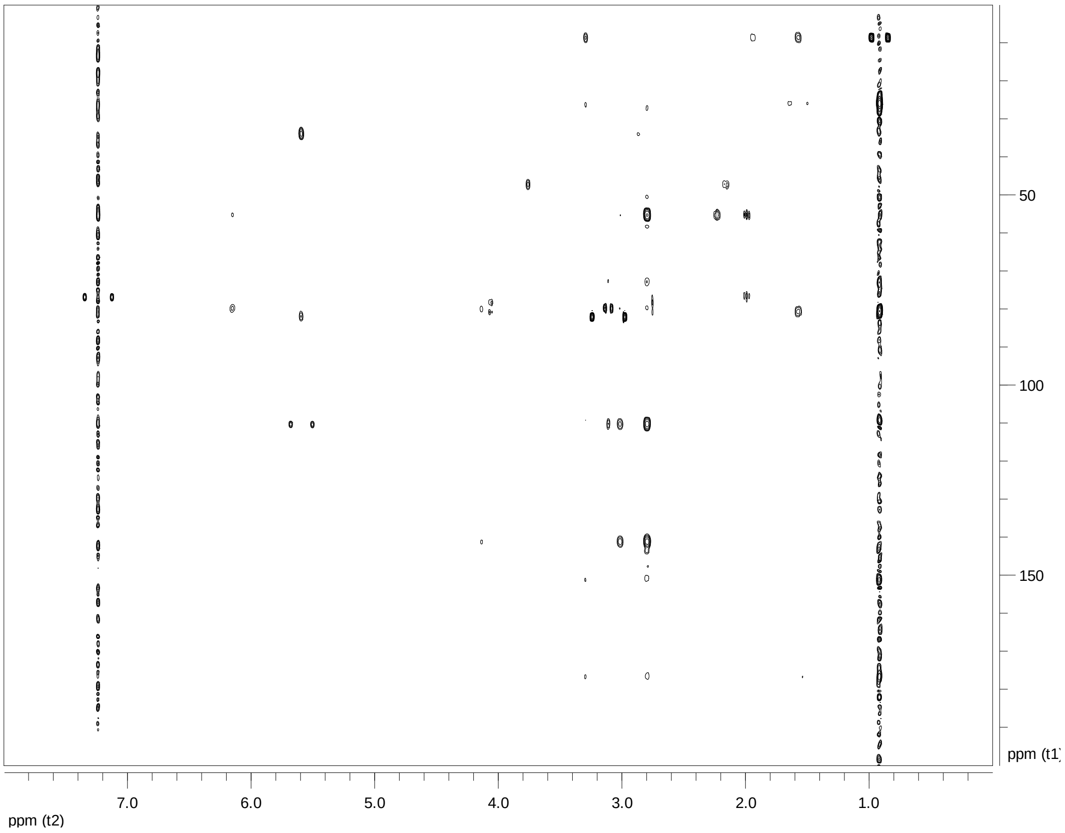

Figure S36. HMBC spectrum of thuwalenyne B (**6**) in $CDCl_3$.

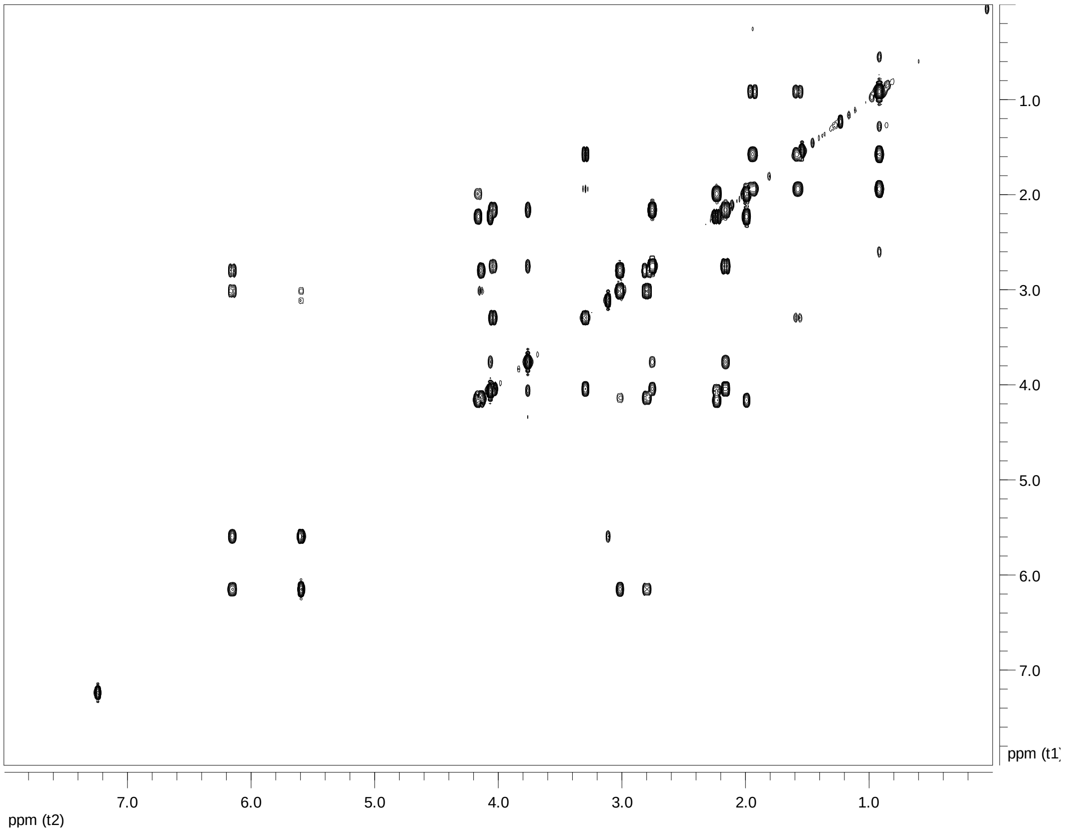

Figure S37. COSY spectrum of thuwalenyne B (**6**) in $CDCl_3$.

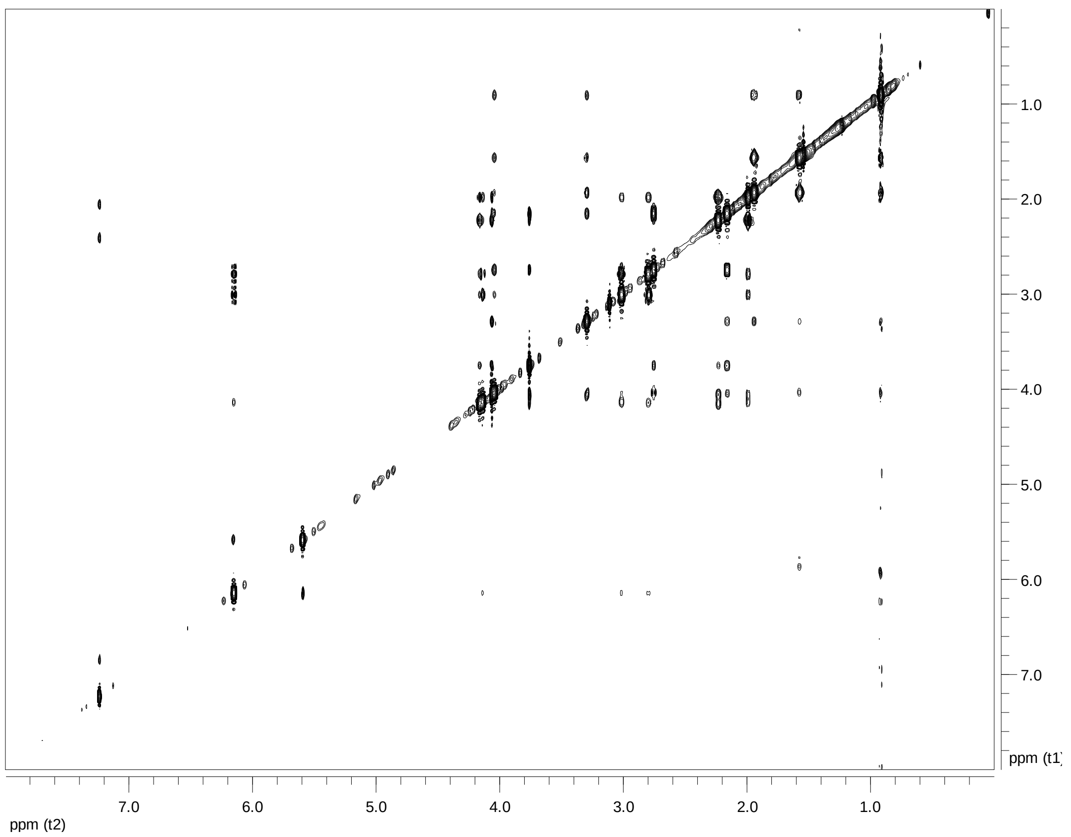

Figure S38. NOESY spectrum of thuwalenyne B (**6**) in $CDCl_3$.

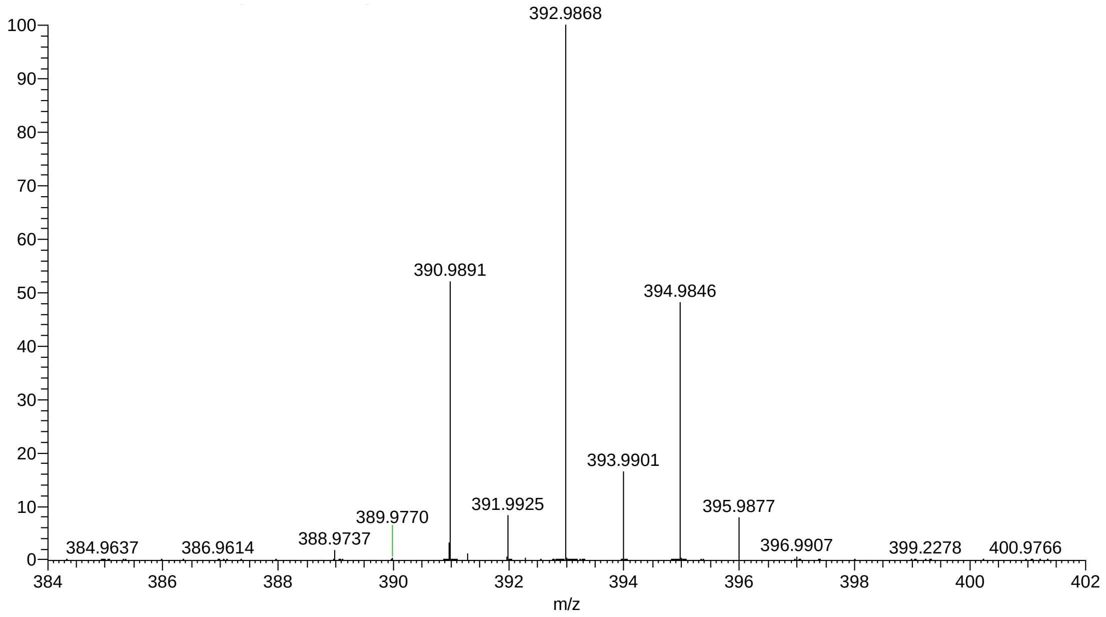

Figure S39. HR-APCIMS spectrum of thuwalenyne B (**6**).

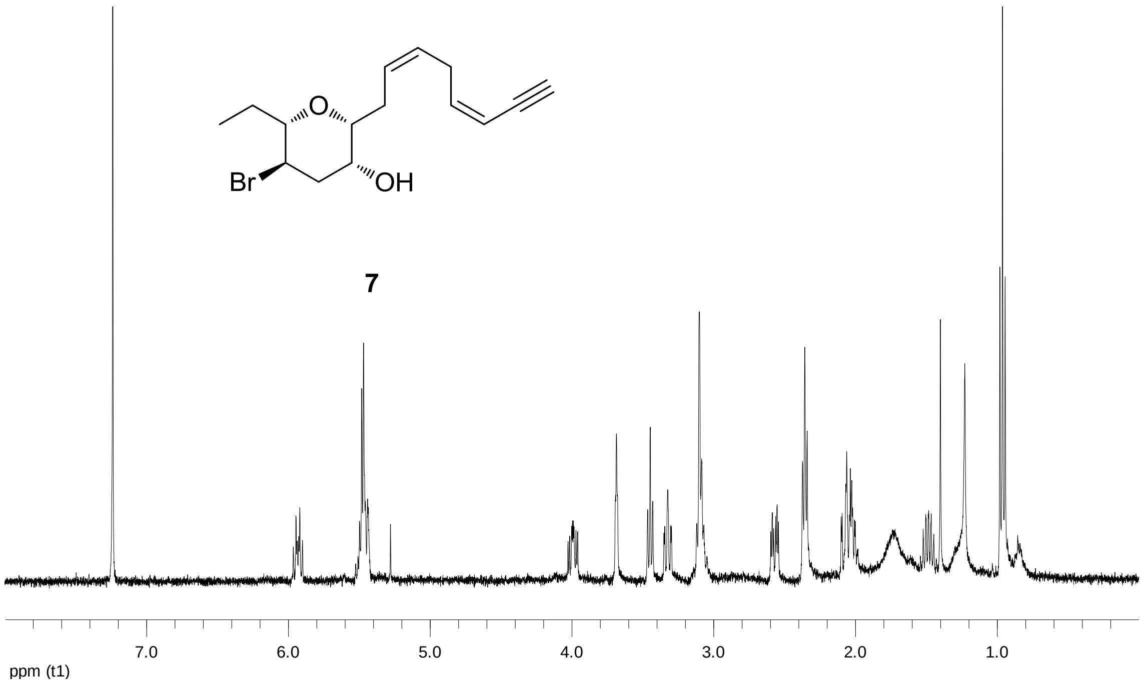

Figure S40. ^{1}H NMR spectrum of thuwalenyne C (**7**) in $CDCl_3$.

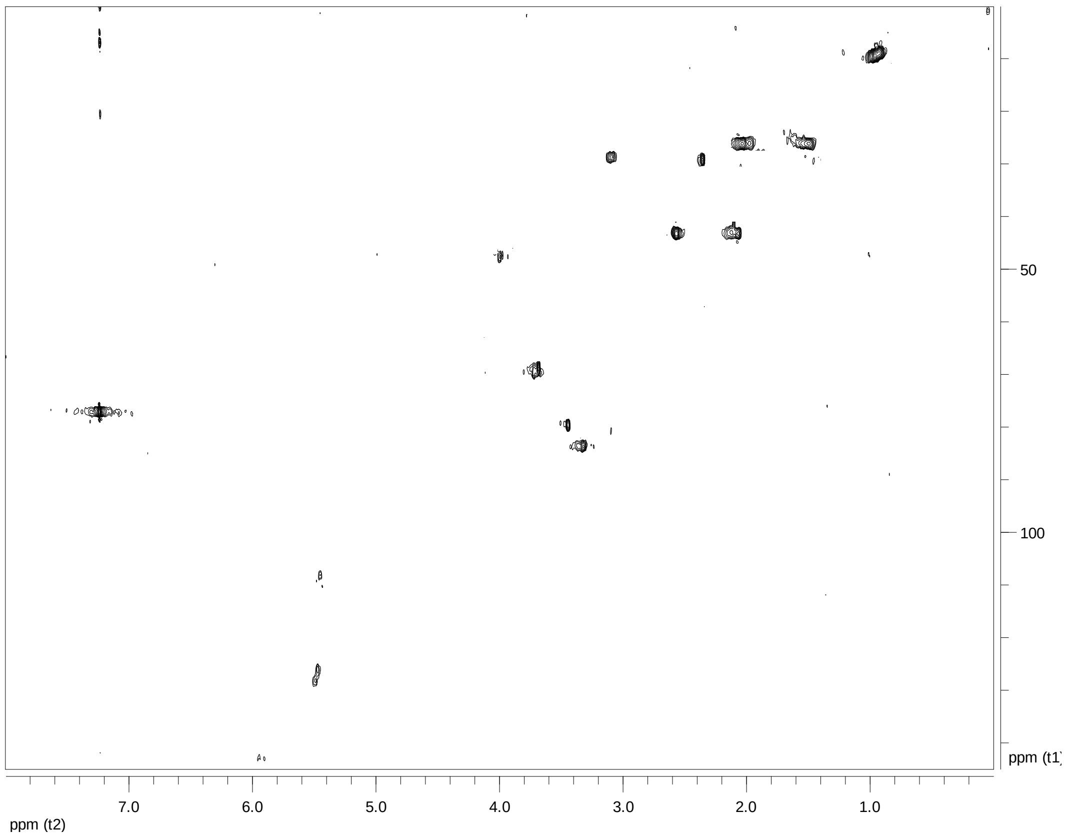

Figure S41. HSQC spectrum of thuwalenyne C (**7**) in $CDCl_3$.

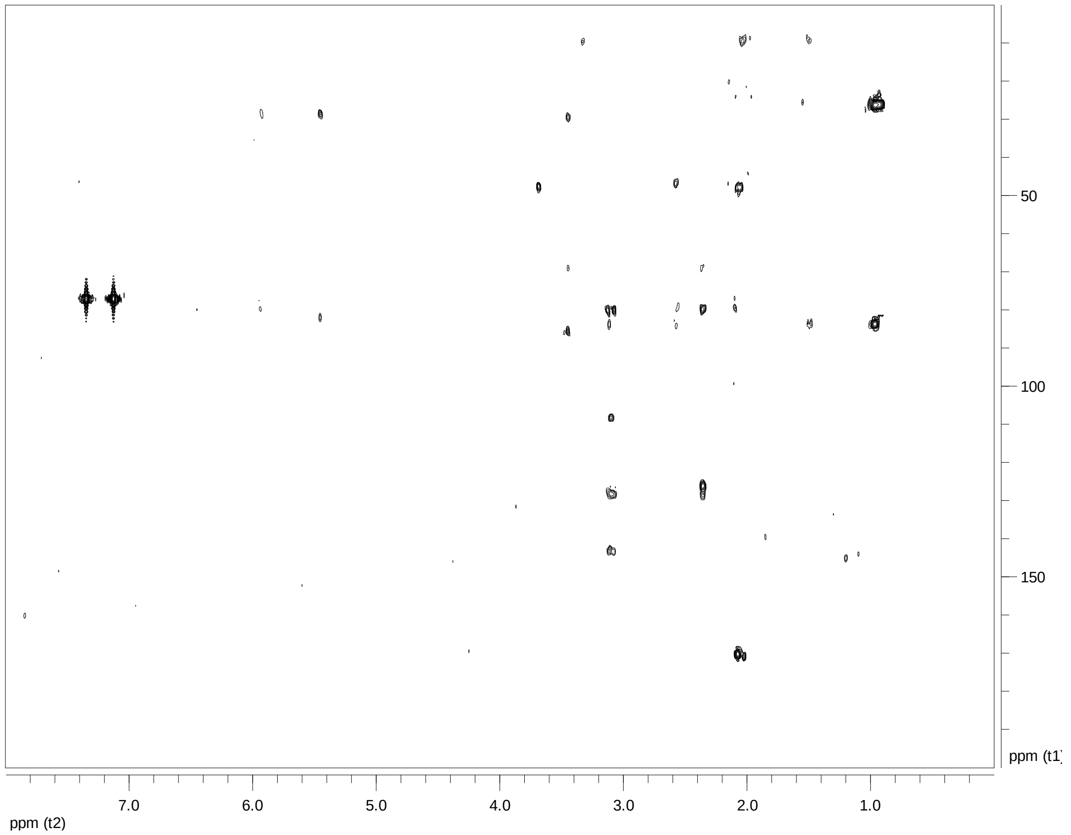

Figure S42. HMBC spectrum of thuwalenyne C (**7**) in $CDCl_3$.

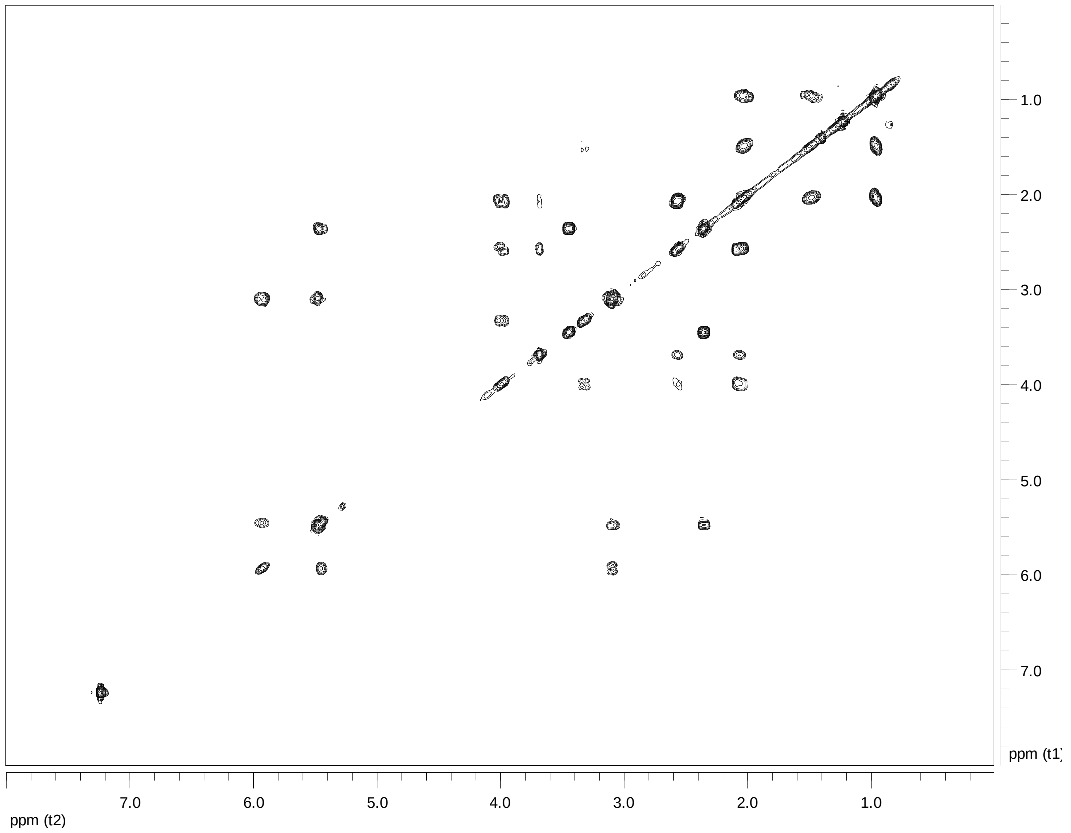

Figure S43. COSY spectrum of thuwalenyne C (**7**) in $CDCl_3$.

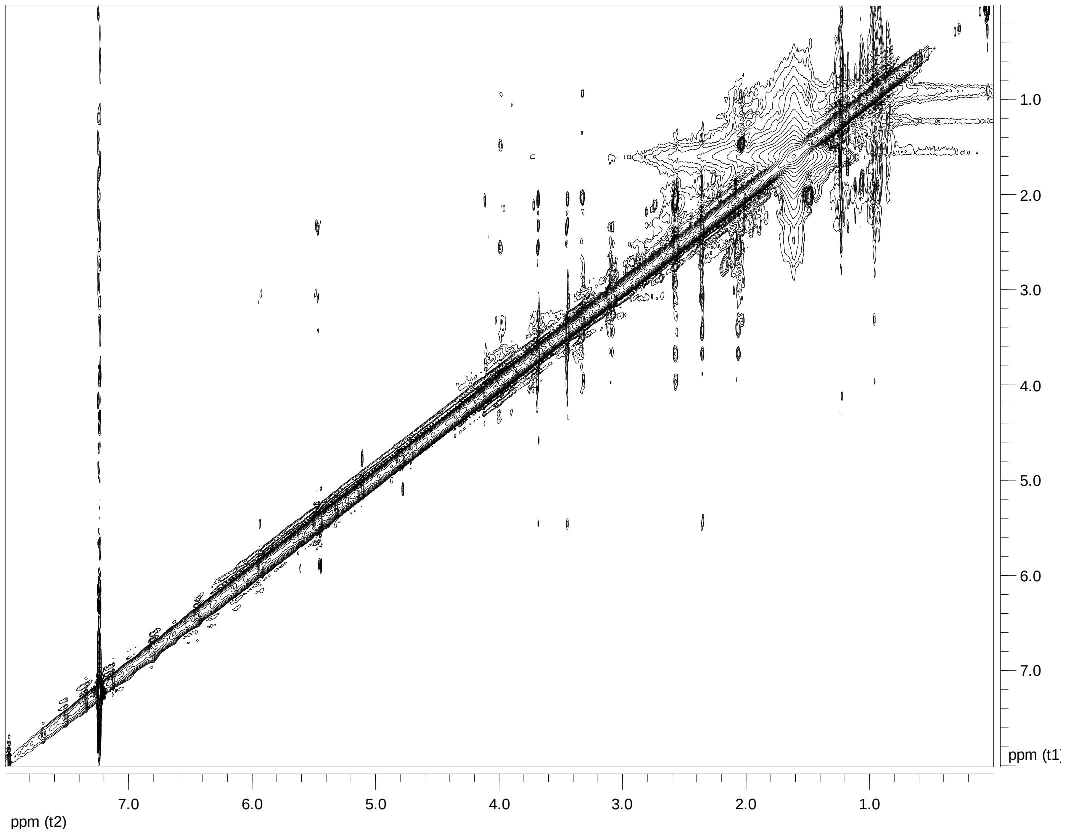

Figure S44. NOESY spectrum of thuwalenyne C (**7**) in $CDCl_3$.

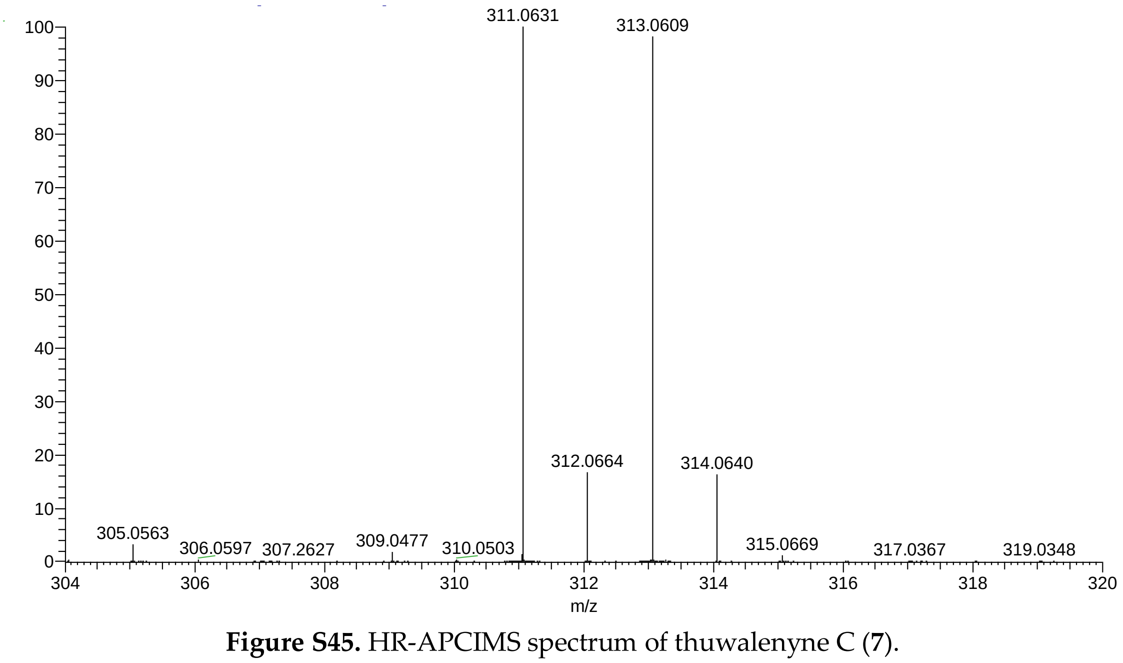

Figure S45. HR-APCIMS spectrum of thuwalenyne C (**7**).

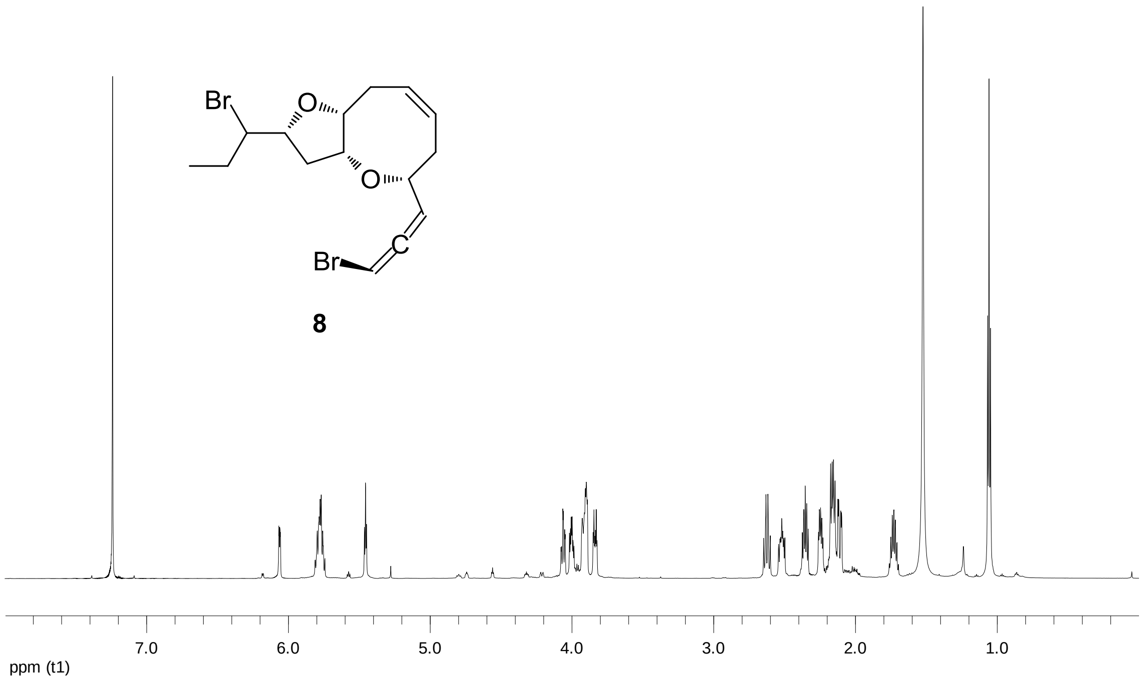

Figure S46. ^{1}H NMR spectrum of thuwalallene E (**8**) in $CDCl_3$.

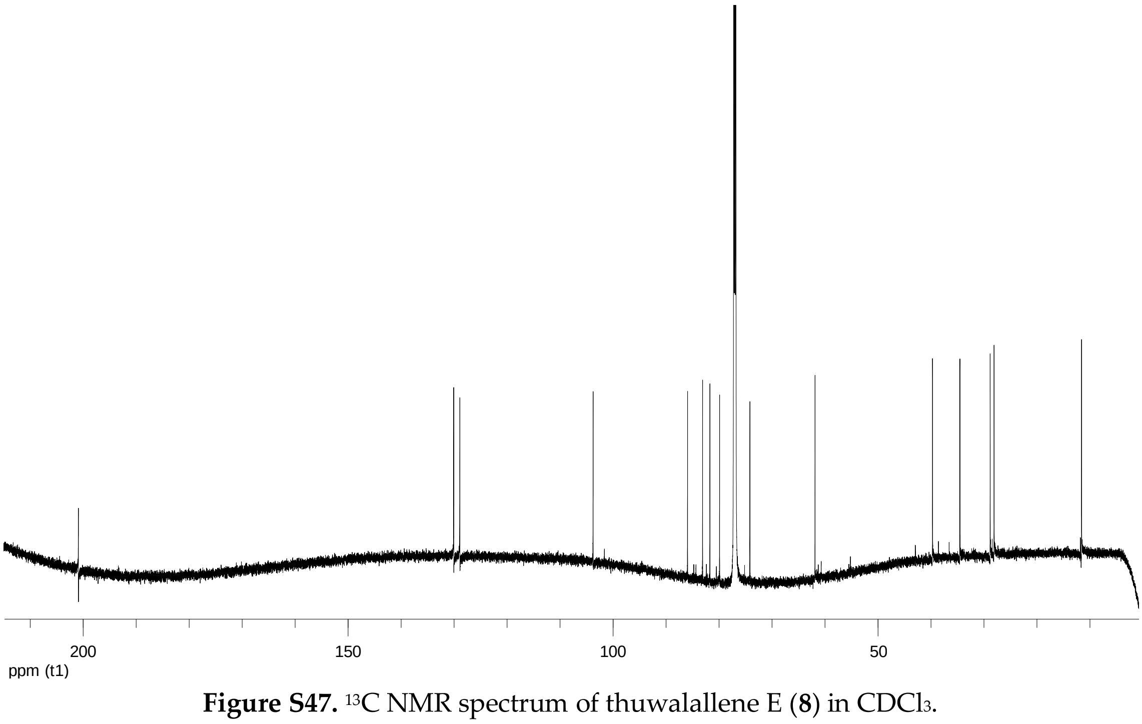

Figure S47. ^{13}C NMR spectrum of thuwalallene E (**8**) in $CDCl_3$.

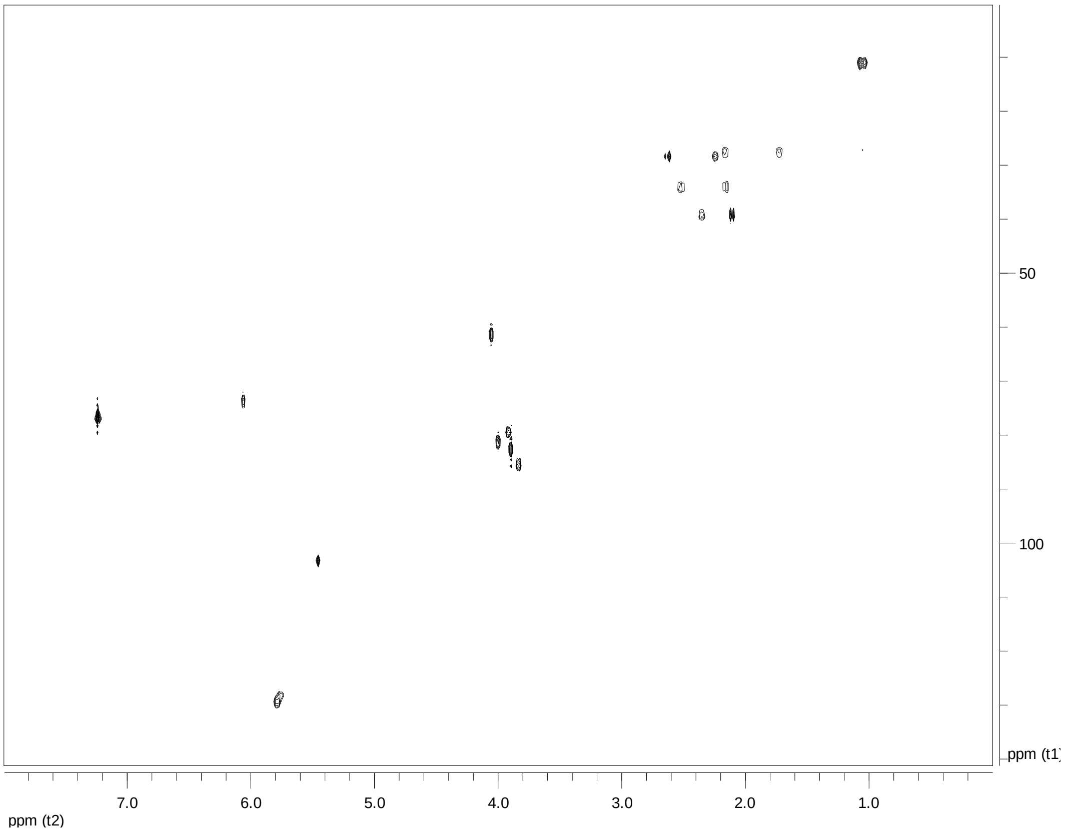

Figure S48. HSQC spectrum of thuwalallene E (**8**) in $CDCl_3$.

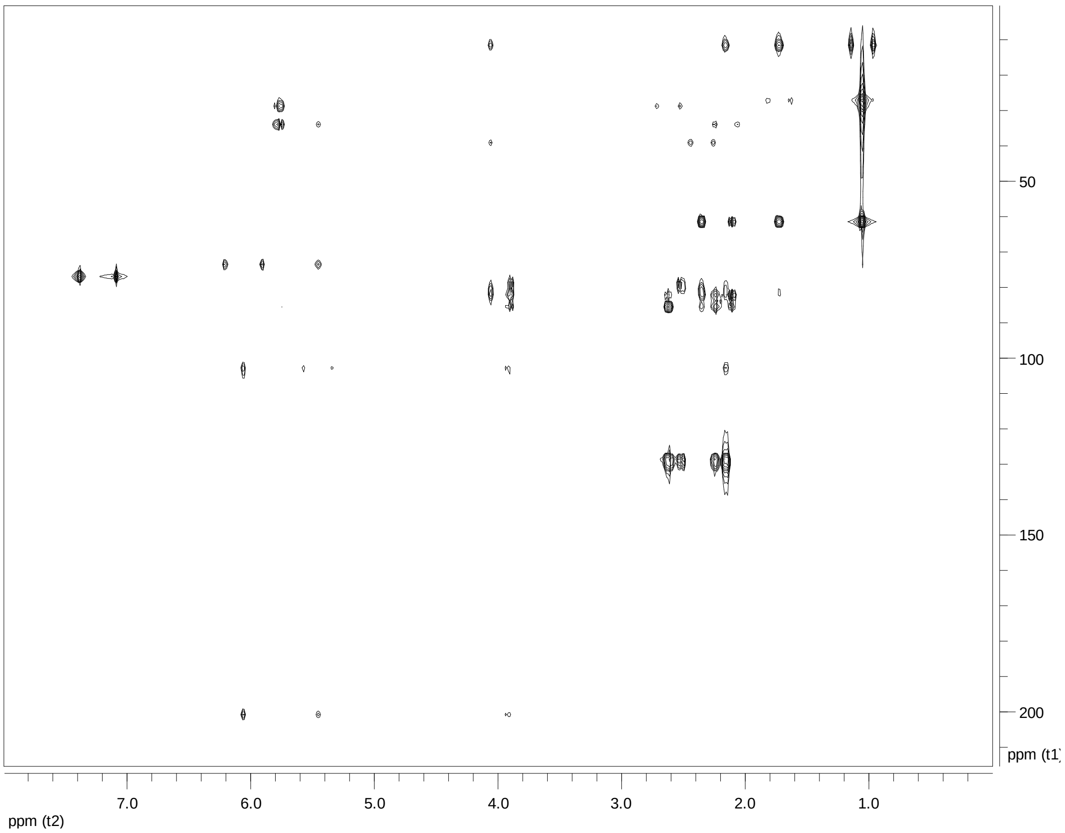

Figure S49. HMBC spectrum of thuwalallene E (**8**) in $CDCl_3$.

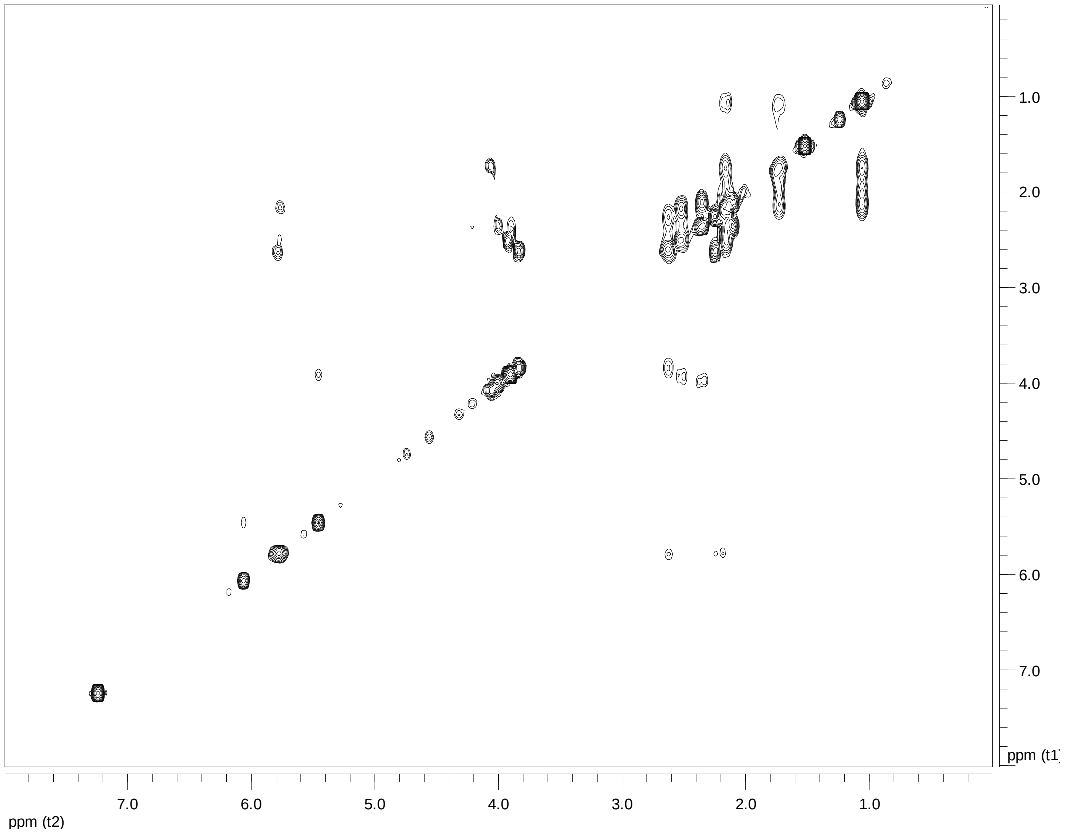

Figure S50. COSY spectrum of thuwalallene E (**8**) in $CDCl_3$.

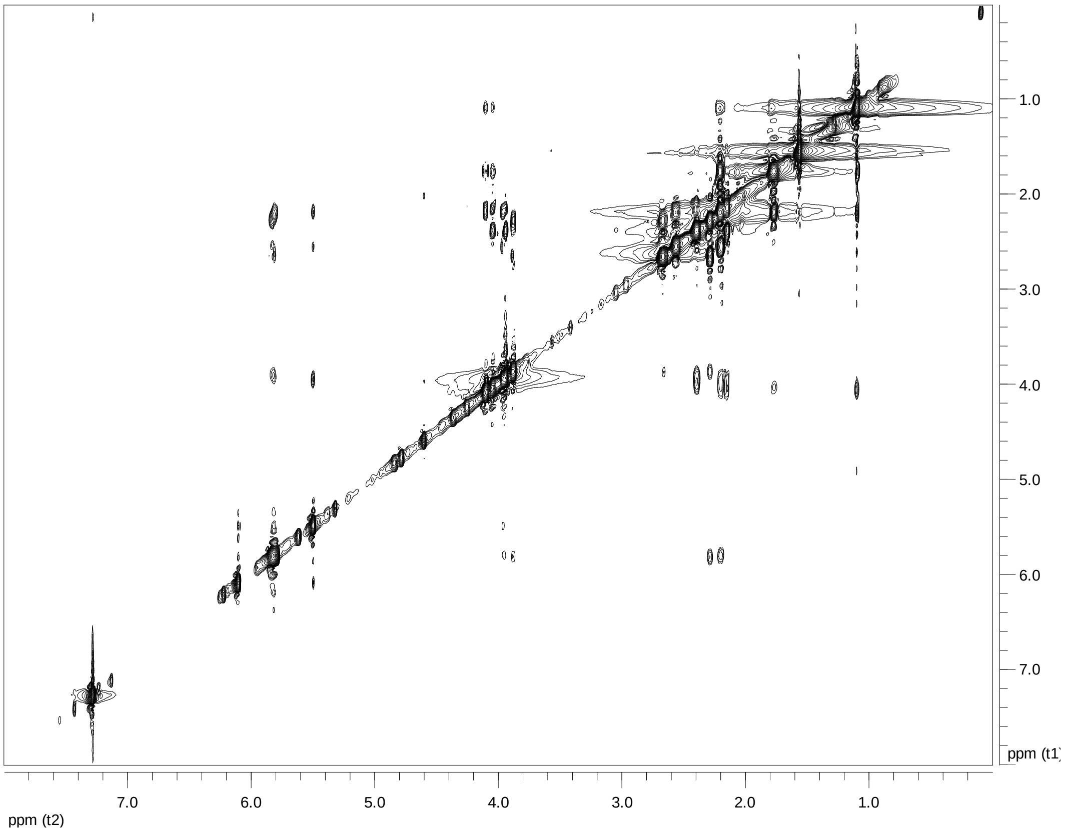

Figure S51. NOESY spectrum of thuwalallene E (**8**) in $CDCl_3$.

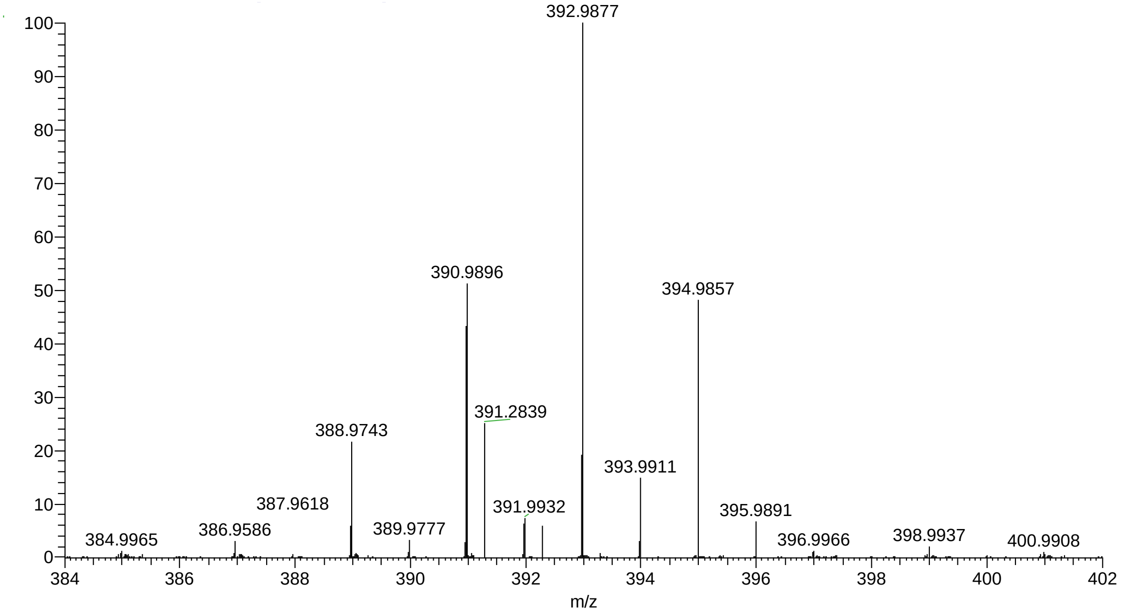

Figure S52. HR-APCIMS spectrum of thuwalallene E (**8**).

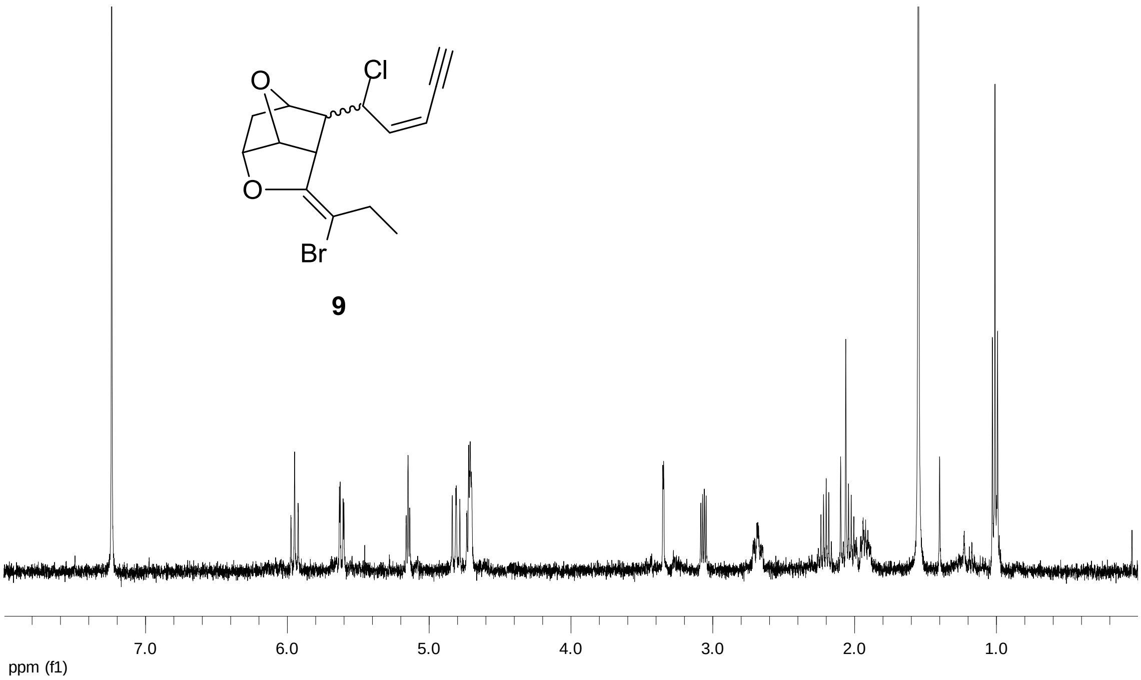

Figure S53. ^{1}H NMR spectrum of *cis*-maneonene D (**9**) in $CDCl_3$.

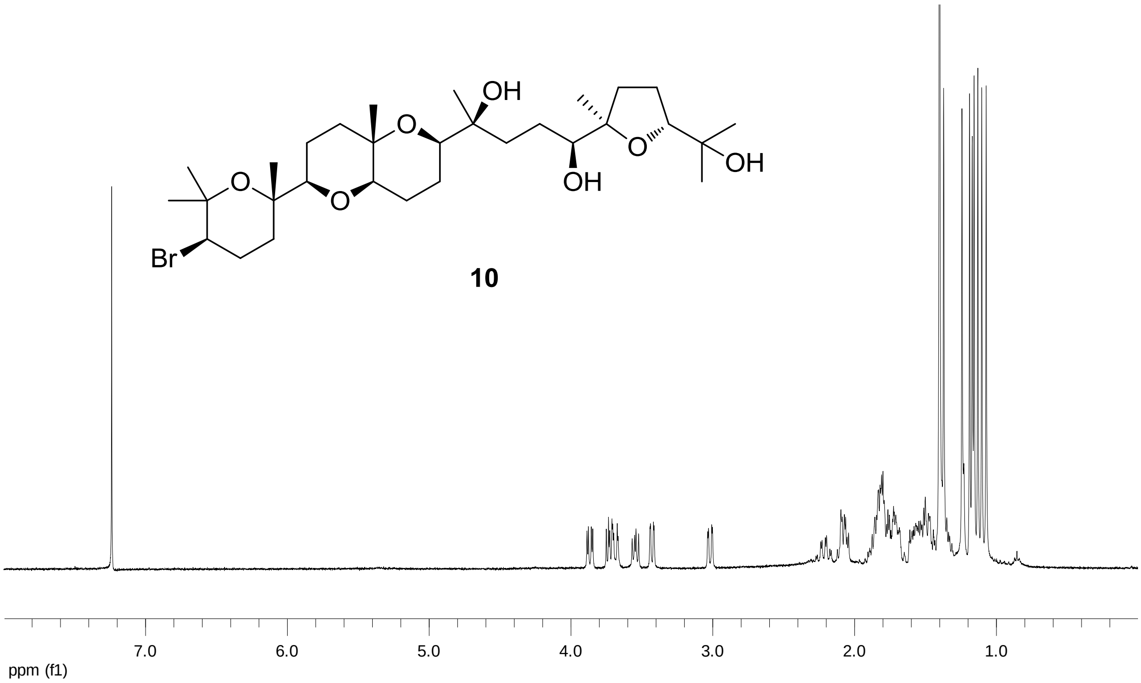

Figure S54. ^{1}H NMR spectrum of thyrsiferol (**10**) in $CDCl_3$.

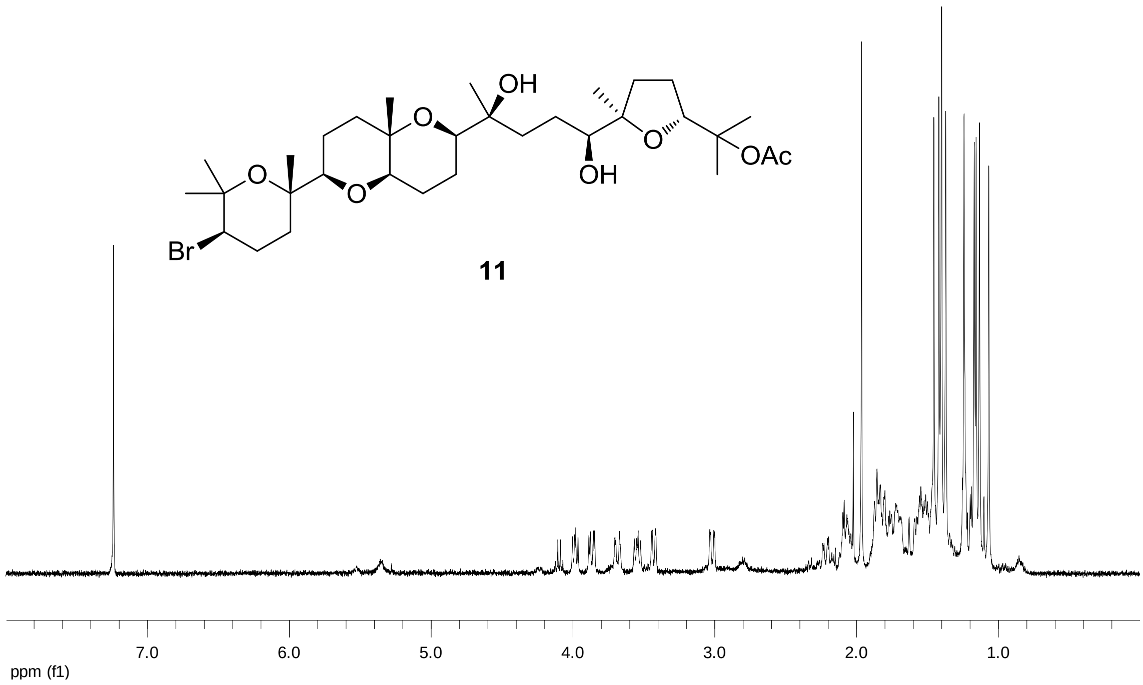

Figure S55. ^{1}H NMR spectrum of 23-acetyl-thyrsiferol (**11**) in $CDCl_3$.
